# Unearthing the Ecology of Soil Microorganisms Using a High Resolution DNA-SIP Approach to Explore Cellulose and Xylose Metabolism in Soil

**DOI:** 10.3389/fmicb.2016.00703

**Published:** 2016-05-12

**Authors:** Charles Pepe-Ranney, Ashley N. Campbell, Chantal N. Koechli, Sean Berthrong, Daniel H. Buckley

**Affiliations:** ^1^School of Integrative Plant Sciences, Cornell UniversityIthaca, NY, USA; ^2^Department of Biological Sciences, Butler UniversityIndianapolis, IN, USA

**Keywords:** stable isotope probing, carbon cycle, decomposition, verrucomicrobia, cellulose, soil, trophic, DNA-SIP

## Abstract

We explored microbial contributions to decomposition using a sophisticated approach to DNA Stable Isotope Probing (SIP). Our experiment evaluated the dynamics and ecological characteristics of functionally defined microbial groups that metabolize labile and structural C in soils. We added to soil a complex amendment representing plant derived organic matter substituted with either ^13^C-xylose or ^13^C-cellulose to represent labile and structural C pools derived from abundant components of plant biomass. We found evidence for ^13^C-incorporation into DNA from ^13^C-xylose and ^13^C-cellulose in 49 and 63 operational taxonomic units (OTUs), respectively. The types of microorganisms that assimilated ^13^C in the ^13^C-xylose treatment changed over time being predominantly *Firmicutes* at day 1 followed by *Bacteroidetes* at day 3 and then *Actinobacteria* at day 7. These ^13^C-labeling dynamics suggest labile C traveled through different trophic levels. In contrast, microorganisms generally metabolized cellulose-C after 14 days and did not change to the same extent in phylogenetic composition over time. Microorganisms that metabolized cellulose-C belonged to poorly characterized but cosmopolitan soil lineages including *Verrucomicrobia, Chloroflexi*, and *Planctomycetes*.

## Introduction

Soils worldwide contain 2300 Pg of carbon (C) which accounts for nearly 80% of the C present in the terrestrial biosphere (Batjes, 1996; Amundson, 2001). Soil microorganisms drive C flux through the terrestrial biosphere and C respiration by soil microorganisms produces annually tenfold more CO_2_ than fossil fuel emissions (Chapin, 2002). Despite the contribution of microorganisms to global C flux, many global C models ignore the diversity of microbial physiology (Six et al., 2006; Allison et al., 2010; Treseder et al., 2011) and we still know little about the ecophysiology of soil microorganisms. Characterizing the ecophysiology of microbes that mediate C decomposition in soil has proven difficult due to their overwhelming diversity. Such knowledge should assist the development and refinement of global C models (Neff and Asner, 2001; Bradford et al., 2008; McGuire and Treseder, 2010; Wieder et al., 2013).

Though microorganisms mediate 80–90% of the soil C-cycle (Coleman and Crossley, 1996; Nannipieri et al., 2003), and microbial community composition can account for significant variation in C mineralization (Strickland et al., 2009), terrestrial C-cycle models rarely consider the community composition of soils (Zak et al., 2006; Reed and Martiny, 2007). Variation in microbial community composition can be linked effectively to rates of soil processes when diagnostic genes for specific functions are available (e.g., nitrogen fixation; Hsu and Buckley, 2009). However, the lack of diagnostic genes for describing soil-C transformations has limited progress in characterizing the contributions of individual microorganisms to decomposition. Remarkably, we still lack basic information on the physiology and ecology of the majority of organisms that live in soils. For example, contributions to soil processes remain uncharacterized for cosmopolitan bacterial phyla in soil such as *Acidobacteria, Chloroflexi, Planctomycetes*, and *Verrucomicrobia*. These phyla combined can comprise 32% of soil microbial communities (based on surveys of the SSU rRNA genes in soil; Buckley and Schmidt, 2002; Janssen, 2006).

To predict whether and how biogeochemical processes vary in response to microbial community structure, it is necessary to characterize functional niches within soil communities. Functional niches defined on the basis of microbial physiological characteristics have been successfully incorporated into biogeochemical process models (e.g., Wieder et al., 2013; Kaiser et al., 2014). In some C-cycle models physiological parameters such as growth rate and substrate specificity are used to define functional niche behavior (Wieder et al., 2013). However, it is challenging to establish the phylogenetic breadth of functional traits. Functional traits are often inferred from the distribution of diagnostic genes across genomes (Berlemont and Martiny, 2013) or from the physiology of isolates cultured on laboratory media (Martiny et al., 2013). For instance, the wide distribution of the glycolysis operon in microbial genomes is interpreted as evidence that many soil microorganisms participate in glucose turnover (McGuire and Treseder, 2010). However, the functional niche may depend less on the distribution of diagnostic genes across genomes and more on life history traits that allow organisms to compete for a given substrate as it occurs in the environment. For instance, rapid resuscitation and fast growth are traits that could allow certain microorganisms to compete effectively for glucose in environments that exhibit high temporal variability. Alternatively, metabolic efficiency and slow growth rates could be traits that allow other microbes to compete effectively for glucose in environments characterized by low temporal variability in glucose supply. These different competitive strategies would not be apparent from genome analysis, or when strains are grown in isolation. Hence, life history traits, rather than genomic capacity for a given pathway, are likely to constrain the diversity of microbes that metabolize a given C source in the soil under a given set of conditions. Therefore, to generate an understanding of functional niche as it relates to biogeochemical processes in soils it is important to characterize microbial functional traits as they occur *in situ* or in microcosm experiments.

Nucleic acid stable-isotope probing (SIP) links genetic identity and activity without the need of diagnostic genetic markers or cultivation and has expanded our knowledge of microbial processes (Chen and Murrell, 2010). Nucleic acid SIP has notable complications, however, including the need to add large amounts of labeled substrate (Radajewski et al., 2000), label dilution resulting in partial labeling of nucleic acids (Radajewski et al., 2000), the potential for cross-feeding and secondary label incorporation (DeRito et al., 2005a), and variation in genome G + C content (Buckley et al., 2007). As a result, most applications of SIP have targeted specialized microorganisms [for instance, methylotrophs (Lueders et al., 2004b), syntrophs (Lueders et al., 2004a), or microorganisms that target pollutants (DeRito et al., 2005b)]. Exploring the soil-C cycle with SIP has proven to be more challenging because SIP has lacked the resolution necessary to characterize the specific contributions of individual microbial groups to the decomposition of plant biomass. High throughput DNA sequencing technology, however, improves the resolving power of SIP (Aoyagi et al., 2015). It is now possible to use far less isotopically labeled substrate resulting in more environmentally realistic experimental conditions. It is also possible to sequence SSU rRNA genes from numerous density gradient fractions across multiple samples thereby increasing the resolution of a typical nucleic acid SIP experiment (Verastegui et al., 2014). With this improved resolution the activity of more soil microorganisms can be assessed. Further, since microbial activities can be more comprehensively assessed, we can begin to determine the ecological properties of functional groups defined by a specific activity in a DNA-SIP experiment. We have employed such a high resolution DNA stable isotope probing approach to explore the assimilation of both xylose and cellulose into bacterial DNA in an agricultural soil.

We added to soil a complex amendment representative of organic matter derived from fresh plant biomass. All treatments received the same amendment but the identity of isotopically labeled substrates was varied between treatments. Specifically, we set up a control treatment where all components were unlabeled, a treatment with ^13^C-xylose instead of unlabeled xylose, and a treatment with ^13^C-cellulose instead of unlabeled cellulose. Soil was sampled at days 1, 3, 7, 14, and 30 and we identified microorganisms that assimilated ^13^C into DNA at each point in time. We designed the experiment to identify soil bacteria that metabolize easily mineralizable C (xylose) and structural C (cellulose) components of plant derived organic matter, to characterize aspects of their ecological niche, and to characterize the temporal dynamics of xylose and cellulose metabolism in soil.

## Methods

### Soil collection and preparation

We collected soils from an organically managed agricultural field in Penn Yan, New York. The agricultural field site has been described previously (Berthrong et al., 2013). Soils were Honoeye/Lima, a silty clay loam on calcareous bedrock, neutral pH, with 12.15 (± s.d. 0.78) mg C g^−1^ dry soil and 1.16 (± s.d. 0.13) mg N g^−1^ dry soil (Berthrong et al., 2013). On November 21, 2011, twelve soil cores (5 cm diameter × 10 cm depth) were collected in duplicate from six different sampling locations in the field using a slide hammer bulk density sampler (coordinates: (1) N 42° 40.288′ W 77° 02.438′, (2) N 42° 40.296′ W 77° 02.438′, (3) N 42° 40.309′ W 77° 02.445′, (4) N 42° 40.333′ W 77° 02.425′, (5) N 42° 40.340′ W 77° 02.420′, (6) N 42° 40.353′ W 77° 02.417′). Soils were sieved (2 mm), homogenized, distributed into flasks (10 g in each 250 ml flask, *n* = 34) and equilibrated at room temperature for 2 weeks until the respiration rate normalized (as determined by GCMS measurement of headspace CO_2_). Sieving causes a transient increase in soil respiration (Datta et al., 2014) and pre-incubation ensures that this labile organic matter is consumed and/or stabilized prior to the beginning of the experiment.

### Soil microcosms

We amended soils with a complex amendment representing plant derived organic matter substituted with either ^13^C-xylose or ^13^C-cellulose to represent easily mineralizable and structural C pools derived from abundant components of plant biomass. By mass the amendment contained 38% cellulose, 23% lignin (alkali extracted, Sigma), 20% xylose, 3% arabinose, 1% galactose, 1% glucose, 0.5% mannose, 10.6% amino acids (Teknova C9795), and 2.9% Murashige Skoog basal salt mixture (which contains macro and micro-nutrients associated with plant biomass; Sigma Aldrich M5524). The amendment had a C:N ratio of 10. This mixture approximates the molecular composition of switchgrass biomass with hemicellulose replaced by its constituent monomers (David and Ragauskas, 2010; Yan et al., 2010). Cellulose (2 mg cellulose g^−1^ d.w. soil) and lignin (1.2 mg lignin g^−1^ d.w. soil) were uniformly distributed over the soil surface as a powder and the remaining constituents were added in solution in a volume of 0.12 ml g^−1^ d.w. soil. The volume of liquid was determined in relation to soil moisture to achieve 50% water holding capacity. Water holding capacity of 50% was chosen, in relation to the texture for this soil, to achieve ~70% water filled pore space, which is the optimal water content for respiration (Linn and Doran, 1984). The total amendment was added to soil at 2.9 mg C g^−1^ soil dry weight (d.w.) which is representative of natural concentrations in soil during early phases of decomposition (Schneckenberger et al., 2008). The C in the amendment comprised 19 % of the total C in the soil. The cellulose-C (0.88 mg C g^−1^ soil d.w.) and xylose-C (0.42 mg C g^−1^ soil d.w.) in the amendment comprised 6 and 3% of the total C in the soil, respectively.

We set up three parallel treatments varying the isotopically labeled component in each treatment (see Figure S1 for outline of experimental design). The treatments were: (1) ^12^C Control Plant Simulant (12CCPS) which included only unlabeled components, (2) ^13^C-Cellulose Plant Simulant (13CCPS) which included ^13^C-cellulose (96 atom % ^13^C, synthesized as described below) instead of unlabeled cellulose, and (3) ^13^C-Xylose Plant Simulant (13CXPS) which included ^13^C_5_-D-xylose (98 atom % ^13^C, Sigma Aldrich) instead of unlabeled xylose. A total of 12 microcosms were established for the ^13^C-xylose and control treatments and 10 for the ^13^C-cellulose treatment. Microcosms were sampled destructively at days 1 (control and xylose only), 3, 7, 14, and 30 and soils were stored at −80°C until nucleic acid extraction. Isotopic analysis was performed at the Cornell University Stable Isotope Laboratory. While microcosm experiments are useful for evaluating microbial activity under defined experimental conditions, results from such experiments need to be verified in the field before drawing conclusions about microbial activity in the natural environment.

### Cellulose production

Bacterial cellulose was produced by *Gluconoacetobacter xylinus* grown in Heo and Son (2002) minimal media (HS medium) made with 0.1% glucose and without inositol. For the production of ^13^C-cellulose, ^13^C_6_-D-glucose, 99 atom % ^13^C (Cambridge Isotope) was used. Cellulose was produced in 1L Erlenmeyer flasks containing 100 mL HS medium inoculated with three colonies of *G. xylinus* grown on HS agar plates. Flasks were incubated statically in the dark at 30°C for 2–3 weeks. Cellulose pellicules were decanted, rinsed with deionized water, suspended in two volumes of 1% Alconox, and then autoclaved. Cellulose pellicules were purified by dialysis for 12 h in 1 L deionized water and dialysis was repeated 10 times. Harvested pellicules were dried overnight (60°C), cut into pieces, and ground using a 5100 Mixer/Mill (SPEX SamplePrep, Metuchen, NJ), and dry sieved to 53–250 μm. The particulate size range was selected to be representative of particulate organic matter in soils.

The purity of ground cellulose was checked by biological assay, Benedict's reducing sugars assay, Bradford assay, and isotopic analysis. *E. coli* is not able to use cellulose as a C source but is capable of growth on a variety of nutrients available in the HS medium. The biological assay consisted of *E. coli* inoculated into minimal M9 media which lacked a C source and was supplemented with either: (1) 0.01% glucose, (2) 2.5 mg purified, ground cellulose, (3) 25 mg purified, ground cellulose, (4) 25 mg purified, ground cellulose and 0.01% glucose. Growth in media was checked by spectrometer (OD_450_). No measurable growth was observed with either 2 mg or 25 mg cellulose, indicating absence of contaminating nutrients that can support growth of *E. coli*. In addition, the presence of 25 mg cellulose did not inhibit the growth of *E. coli* cultures provided with glucose (relative to control), indicating the absence of compounds in the purified cellulose that could inhibit microbial growth (data not shown). Purified cellulose was also assayed for residual proteins and sugars using Bradford and Benedict's assays, respectively. Bradford assay was performed as in Bradford (1976). Ground, purified cellulose contained 6.92 μg protein mg cellulose^−1^(*i.e.*, 99.31% purity). Reducing sugars were not detected in cellulose using Benedict's reducing sugar assay (Benedict, 1909) tested at 10 mg cellulose ml^−1^ (data not shown). Finally, ^13^C-cellulose had an average 96% ± 5 (s.d.) degree of ^13^C labeling as determined by isotopic analysis (UCDavis Stable Isotope Facility).

### Nucleic acid extraction

Nucleic acids were extracted from 0.25 g soil using a modified Griffiths protocol (Griffiths et al., 2000). Cells were lysed by bead beating for 1 min at 5.5 m s^−1^ in 2 mL lysis tubes containing 0.5 g of 0.1 mm diameter silica/zirconia beads (treated at 300°C for 4 h to remove RNAses), 0.5 mL extraction buffer (240 mM Phosphate buffer 0.5% N-lauryl sarcosine), and 0.5 mL phenol-chloroform-isoamyl alcohol (25:24:1) for 1 min at 5.5 m s^−1^. After lysis, 85 μL 5 M NaCl and 60 μL 10% hexadecyltriammonium bromide (CTAB)/0.7 M NaCl were added to lysis tube, vortexed, chilled for 1 min on ice, and centrifuged at 16,000 x g for 5 min at 4°C. The aqueous layer was transferred to a new tube and reserved on ice. To increase DNA recovery, the pellet was back extracted with 85 μL 5 M NaCl and 0.5 mL extraction buffer. The aqueous extract was washed with 0.5 mL chloroform:isoamyl alcohol (24:1). Nucleic acids were precipitated by addition of 2 volumes polyethylene glycol solution (30% PEG 8000, 1.6 M NaCl) on ice for 2 h, followed by centrifugation at 16,000 x g, 4°C for 30 min. The supernatant was discarded and pellets were washed with 1 mL ice cold 70% EtOH. Pellets were air dried, resuspended in 50 μL TE and stored at −20°C. To prepare nucleic acid extracts for isopycnic centrifugation as previously described Buckley et al. (2007), DNA was size selected (>4 kb) using 1% low melt agarose gel and β-agarase I enzyme extraction per manufacturers protocol (New England Biolab, M0392S). Final resuspension of DNA pellet was in 50 μL TE.

### Isopycnic centrifugation and fractionation

We fractionated DNA on density gradients for ^13^C-xylose treatments (days 1, 3, 7, 14, 30), ^13^C-cellulose treatments (days 3, 7, 14, 30), and control treatments (days 1, 3, 7, 14, 30). A total of 5 μg DNA was added to each 4.7 mL CsCl density gradient. Density gradient were composed of 1.69 g ml^−1^ CsCl ml^−1^ in gradient buffer solution (pH 8.0 15 mM Tris-HCl, 15 mM EDTA, 15 mM KCl). Centrifugation was performed at 55,000 rpm 20°C for 66 h using a TLA-110 rotor in a Beckman Coulter Optima MAX-E ultracentrifuge. Fractions of ~100 μL were collected from below by displacing the DNA-CsCl-gradient buffer solution in the centrifugation tube with water using a syringe pump at a flow rate of 3.3 μL s^−1^ (Manefield et al., 2002). Fractions were collected in Acroprep 96 filter plates (part no. 5035, Pall Life Sciences). The refractive index of each fraction was measured using a Reichart AR200 digital refractometer modified as previously described to measure a volume of 5 μL (Buckley et al., 2007). Buoyant density was calculated from the refractive index as previously described Buckley et al. (2007) using the equation ρ = *a*η − *b*, where ρ is the density of the CsCl (g ml^−1^), η is the measured refractive index, and *a* and *b* are coefficient values of 10.9276 and 13.593, respectively, for CsCl at 20°C (Birnie, 1978). The refractive index (Ri) was corrected to account for the Ri of the gradient buffer using the equation: *Ri*_*corrected*_ = *Ri*_*observed*_ − (*Ri*_*buffer*_ − 1.3333). A total of 35 fractions were collected from each gradient and the average density between fractions was 0.0040 g mL^−1^. The DNA was desalted by washing with TE (5X 200 μL) in the Acroprep filter wells. DNA was resuspeneded in 50 μL TE.

### PCR amplification

SSU rRNA genes were amplified from gradient fractions (*n* = 20 per gradient) and from non-fractionated DNA from soil. Barcoded primers consisted of: 454-specific adapter B, a 10 bp barcode, a 2 bp linker (5′-CA-3′), and 806R primer for reverse primer (BA806R); and 454-specific adapter A, a 2 bp linker (5′-TC-3′), and 515F primer for forward primer (BA515F). Each PCR contained 1.25 U μl−1 AmpliTaq Gold (Life Technologies, Grand Island, NY; N8080243), 1X Buffer II (100 mM Tris-HCl, 500 mM KCl, pH 8.3), 2.5 mM MgCl2, 200 μM of each dNTP, 0.5 mg ml^−1^ BSA, 0.2 μM BA515F, 0.2 μM BA806R, and 10 μL of 1:30 DNA template in 25 μl total volume. The PCR conditions were 95°C for 5min followed by 22 cycles of 95°C for 10 s, 53°C for 30 s, and 72°C for 30 s, followed by a final elongation at 72°C for 5 min. Amplification products were checked by 1% agarose gel. Reactions were performed in triplicate and pooled. Amplified DNA was gel purified (1% low melt agarose) using the Wizard SV gel and PCR clean-up system (Promega, Madison, WI; A9281) per manufacturer's protocol. Samples were normalized by SequalPrep normalization plates (Invitrogen, Carlsbad, CA; A10510) or based on PicoGreen DNA quantification and pooled in equimolar concentration. Amplicons were sequenced on Roche 454 FLX system using titanium chemistry at Selah Genomics (Columbia, SC).

### DNA sequence analysis

SSU rRNA gene sequences were initially screened by maximum expected errors at a specific read length threshold (Edgar, 2013). Reads that had more than 0.5 expected errors at a length of 250 nt were discarded. The remaining reads were aligned to the Silva Reference Alignment as provided in the Mothur software package using the Mothur NAST aligner (DeSantis et al., 2006; Schloss et al., 2009). Reads that did not align to the expected region of the SSU rRNA gene were discarded. After expected error and alignment based quality control. The remaining quality controlled reads were annotated using the “UClust” taxonomic annotation framework in QIIME (Caporaso et al., 2010; Edgar, 2010). We used 97% sequence identity cluster seeds from the Silva SSU rRNA database (release 111Ref; Quast et al., 2013) as reference for taxonomic annotation [provided on the QIIME website, (Quast et al., 2013)]. Quality control screening filtered out 344,472 of 1,720,480 sequencing reads leaving 1,376,008 reads for downstream analyses. Reads annotated as “Chlorloplast,” “Eukaryota,” “Archaea,” “Unassigned” or “mitochondria” were culled from the dataset.

Sequences were distributed into OTUs with a centroid based clustering algorithm [i.e., UPARSE; Edgar (2013)]. The centroid selection also included chimera screening (Edgar, 2013). OTU centroids were established at a threshold of 97% sequence identity and non-centroid sequences were mapped back to centroids. Reads that could not be mapped to an OTU centroid at greater than or equal to 97% sequence identity were discarded. OTU centroids were compared (BLAST; Altschul et al., 1990; Camacho et al., 2009) to sequences in “The All-Species Living Tree” project (LTP). The LTP (version 115) is a collection of SSU rRNA gene sequences for named and cultured species of Archaea and Bacteria (Yarza et al., 2008).

We used SSU-Align (Nawrocki et al., 2009; Nawrocki and Eddy, 2013) to align SSU rRNA gene sequences. Columns in the alignment that were aligned with poor confidence (<95% of characters had posterior probability >95%) were not considered when building the phylogenetic tree leaving a multiple sequence alignment of 216 columns. Additionally, the alignment was trimmed to coordinates such that all sequences in the alignment began and ended at the same positions. FastTree was used with default parameters to build the phylogeny (Price et al., 2010).

### Statistical analyses

NMDS ordination was performed on weighted Unifrac (Lozupone and Knight, 2005) distances between samples. The Phyloseq (McMurdie and Holmes, 2013) wrapper for Vegan (Oksanen et al., 2015; both R packages) was used to compute sample values along NMDS axes. The “Adonis” function in Vegan was used to perform permutational MANOVA (default parameters; Anderson, 2001).

We used DESeq2 (R package), an RNA-Seq differential expression statistical framework (Love et al., 2014), to identify OTUs that were enriched in high density gradient fractions from ^13^C-treatments relative to corresponding gradient fractions from control treatments [for review of RNA-Seq differential expression statistics applied to microbiome OTU count data see (McMurdie and Holmes, 2014)]. Briefly, DESeq2 includes several features that enable robust estimates of standard error in addition to reliable ranking of logarithmic fold change (LFC; i.e., gamma-Poisson regression coefficients) in OTU relative abundance even with low count OTUs. Figures S2, S3 demonstrate raw data for one example responder and non-responder OTU, respectively. Responders increase in relative abundance in the high density fractions relative to control due to ^13^C-labeling of their DNA. As our data is compositional, unlabeled OTUs often had consistent *relative* abundance across the density gradients indicating the OTU DNA concentration across the gradient mirrored that of the total DNA concentration. For each OTU, we calculated LFC and corresponding standard errors for enrichment in high density gradient fractions of ^13^C treatments relative to control. We define “high density gradient fractions” as gradient fractions whose density falls between 1.7125 and 1.755 g ml^−1^. Subsequently, a one-sided Wald test was used to statistically assess LFC values. The user-defined null hypothesis was that LFC was less than one standard deviation greater than the mean of all LFC values. We independently filtered OTUs prior to multiple comparison corrections on the basis of sparsity eliminating OTUs that failed to appear in at least 45% of high density fractions for a given comparison. *P*-values were adjusted for multiple comparisons using the Benjamini and Hochberg method (Benjamini and Hochberg, 1995). We selected a false discovery rate of 10% to denote statistical significance.

We identified OTUs that changed in relative abundance over time again using gamma-poisson regression (R package DESeq2, Love et al., 2014). Specifically, we used day as an ordered factor as the regressor with LFC of the relative abundance in non-fractionated DNA as the outcome in the general linear model. We used the default DESeq2 base mean independent filtering and disabled the Cook's cutoff outlier detection. The null model was that abundance did not change with time and we assessed significance at a false discovery rate of 10%.

We estimated the *rrn* copy number for each OTU as described; i.e., we used the code and reference information provided by the authors (Kembel et al., 2012) directly. In brief, OTU centroid sequences were inserted into a reference SSU rRNA gene phylogeny (Matsen et al., 2010) from organisms of known *rrn* copy number. The *rrn* copy number was then inferred from the phylogenetic placement in the reference phylogeny.

The Net Relatedness Index (NRI) and Nearest Taxon Index (NTI) were calculated using the “picante” R package (Kembel et al., 2010). We used the “independentswap” null model for phylogenetic distribution. Briefly, the NRI and NTI evaluate phylogenetic clustering against a null model for the distribution of a trait in a phylogeny (Webb, 2000). The NRI and NTI values are z-scores and thus the greater the magnitude of the NRI/NTI, the stronger the evidence for clustering (positive values) or overdispersion (negative values). NRI assesses basal clustering whereas the NTI assesses terminal clustering (Evans and Wallenstein, 2014). The consenTRAIT clade depth for xylose and cellulose responders was calculated using R code from the original publication describing the metric (Martiny et al., 2013) which employs the R “adephylo” package (Jombart and Dray, 2010). The consenTRAIT metric is a measure of the average clade depth for a trait in a phylogenetic tree.

We calculated the change in center of mass for buoyant density, $\Delta \hat{BD}$, for the relative abundance of each OTU as the change in an OTU's density profile center of mass (see below) between corresponding control and labeled gradients (Figure S2). DNA buoyant density (BD) increases with atom % ^13^C. Therefore, the magnitude of $\Delta \hat{BD}$ for an OTU in response to ^13^C labeling provides a measure of the degree of isotopic labeling for an OTU. Because all gradients did not span the same density range and gradient fractions cannot be taken at specific density positions, we limited our $\Delta \hat{BD}$ analysis to the density range for which fractions were taken for all gradients. Within this density range we linearly interpolated 20 evenly spaced relative abundance values. The center of mass for an OTU along the density gradient was then the density weighted average where weights were the linearly interpolated relative abundance values. The $\Delta \hat{BD}$ values determined in this way do not represent the isotopic incorporation of any specific strain but rather represent an average for each OTU. This average can be shifted by the ratio of active and inactive strains within each OTU as well as by isotope dilution effects. Furthermore, values based on compositional data can be distorted in comparison to values based on absolute DNA concentration. Hence, $\Delta \hat{BD}$ values calculated in this way provide a qualitative estimate of the extent of labeling (for example, does DNA labeled in response to ^13^C-xylose additions contain on average more ^13^C than DNA labeled in response to the addition ^13^C-cellulose) but do not provide precise quantification of isotope incorporation. Note, that these $\Delta \hat{BD}$ values were not used to identify OTUs that incorporated ^13^C into DNA (for the identification of ^13^C-responsive OTUs, see instead LFC calculations as described above).

All code to take raw SSU rRNA gene sequencing reads to final publication figures and through all presented analyses is located at the following URL: https://github.com/chuckpr/CSIP_succession_data_analysis. DNA sequences are available at the NCBI Short Read Archive (Accession SRP072785).

## Results

We sequenced SSU rRNA gene amplicons from a total of 277 CsCl gradient fractions representing 14 CsCl gradients and 12 non-fractionated DNA samples. The SSU rRNA gene data set contained 1,102,685 total sequences. The average number of sequences per sample was 3816 (s.d. 3629) and 265 samples had over 1000 sequences. We sequenced SSU rRNA gene amplicons from an average of 20 fractions per CsCl gradient (s.d. 0.57). The average density between fractions was 0.0040 g mL^−1^. The sequencing effort recovered a total of 5940 OTUs. 2943 of the total 5940 OTUs were observed in non-fractionated samples. We observed 33 unique phylum and 340 unique genus annotations. Analysis of SSU rRNA gene amplicons from non-fractionated soil DNA (Figure S4) indicated that variance in community composition was mainly driven by time and that, in the absence of SIP, little variance in community composition could be attributed to isotopic treatment or block effects (i.e., bottle to bottle variance was small relative to variance over time).

### Community-level signal of ^13^C-assimilation in relation to substrate and time

**Figure 1:**
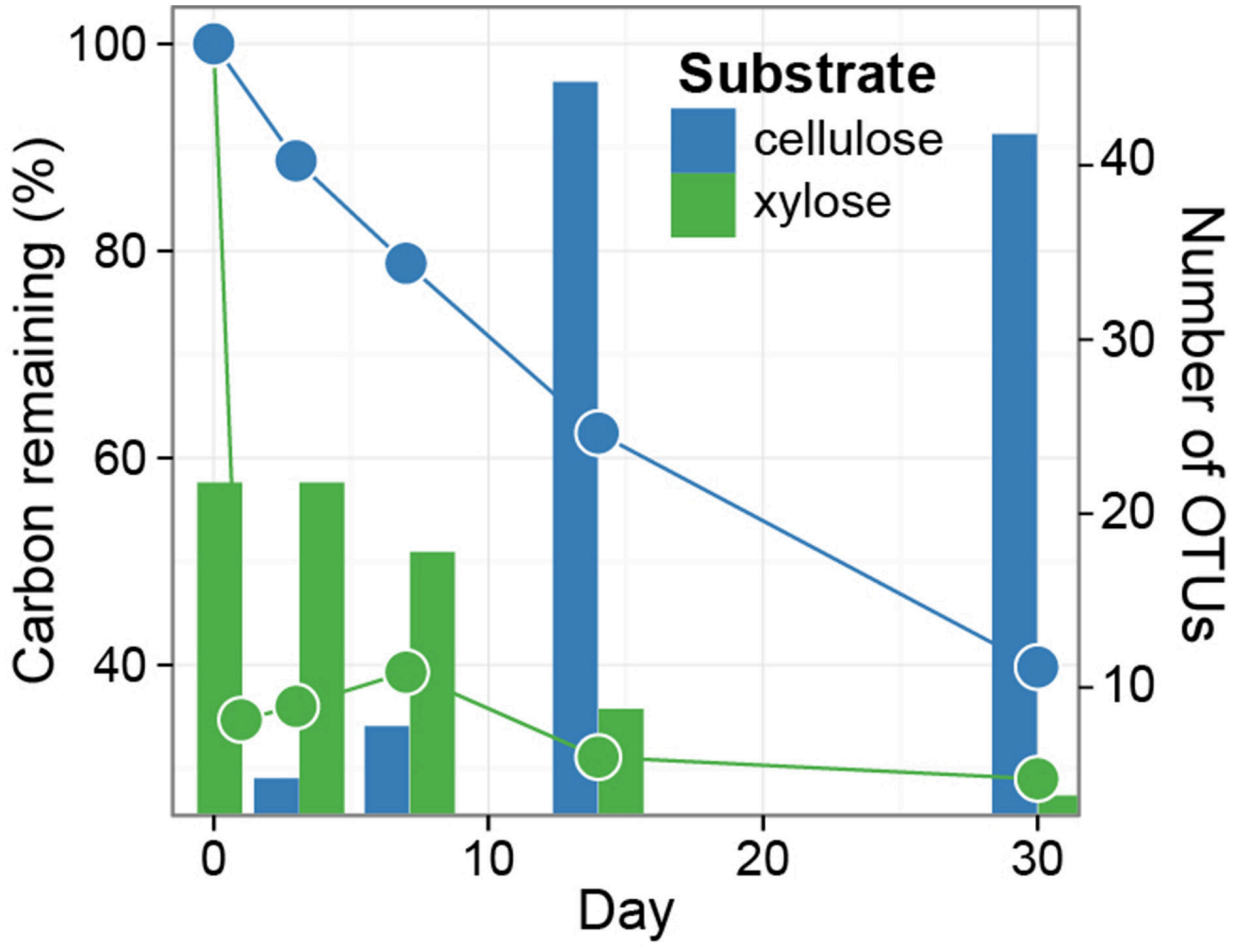
**Distinct dynamics of metabolization are observed for ^13^C-xylose and ^13^C-cellulose, as expected**. Symbols indicate the percentage of added ^13^C that remains in soil over time. Soils were pooled (three samples per time point per treatment) prior to measuring ^13^C-content. The bars indicate enumerate OTUs found by SIP to assimilate ^13^C into DNA (i.e., counts of xylose and cellulose responders) over time (days 1, 3, 7, 14, 30 for xylose, and days 3, 7, 14, 30 for cellulose). Note that ^13^C lost from soil must be lost in gaseous form, and that ^13^C that remains in soil may be either unmetabolized, transformed into a new chemical form, or assimilated into microbial biomass.

The soil microbial community respired 65% of the added xylose within 1 day and 29% of the ^13^C from xylose remained in the soil at day 30 (Figure 1). In contrast, cellulose was respired at an average rate of 18 μg C d^−1^ g^−1^ soil d.w. and 40% of added ^13^C from cellulose remained in the soil at day 30 (Figure 1).

**Figure 2:**
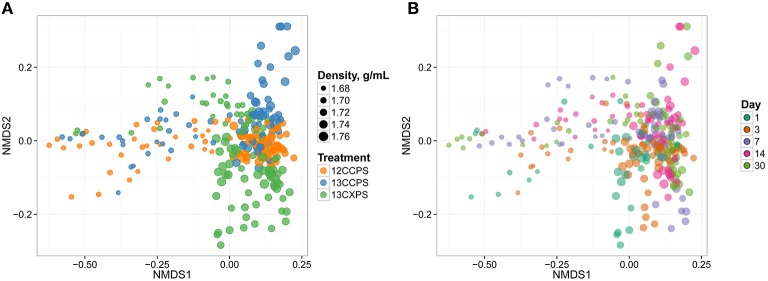
**NMDS ordination of SSU rRNA gene sequence composition in gradient fractions shows that variation between fractions is correlated with fraction density, isotopic labeling, and time**. Dissimilarity in SSU rRNA gene sequence composition was quantified using the weighted UniFrac metric. SSU rRNA gene sequences were surveyed in twenty gradient fractions at each sampling point for each treatment (Figure S1). ^13^C-labeling of DNA is apparent because the SSU rRNA gene sequence composition of gradient fractions from ^13^C and control treatments differ at high density. Each point on the NMDS plot represents one gradient fraction. The size of each point is positively correlated with density and colors indicate the treatment **(A)** or day **(B)**.

We initially assessed assimilation of ^13^C into microbial DNA by comparing the SSU rRNA gene sequence composition of SIP density gradient fractions between ^13^C treatments and the unlabeled control. In the unlabeled control treatment, fraction density represented the majority of the variance in SSU rRNA gene composition (Figure 2). This result is expected because genome G + C content correlates positively with DNA buoyant density and influences SSU rRNA gene composition in gradient fractions (Buckley et al., 2007). In contrast, isotope assimilation into DNA will cause variation in gene sequence composition between corresponding density fractions from controls and labeled treatments. For example, the SSU rRNA gene composition in gradient fractions from the ^13^C-cellulose treatment deviated from corresponding control fractions on days 14 and 30 and this difference was observed only in the high density fractions (>1.7125 g mL^−1^, Figure 2). Likewise, SSU rRNA gene composition in gradient fractions from the ^13^C-xylose treatment also deviated from corresponding control fractions but on days 1, 3, and 7 as opposed to 14 and 30 (Figure 2). The ^13^C-cellulose and ^13^C-xylose treatments also differed from each other in corresponding high density gradient fractions indicating that different microorganisms were labeled across time in these treatments (Figure 2).

We observed further differences in the pattern of isotope incorporation over time for each treatment. For example the SSU rRNA gene sequence composition in the ^13^C-cellulose treatment was similar on days 14 and 30 in corresponding high density fractions indicating similar patterns of isotope incorporation into DNA on these days (Figure 2). In contrast, in the ^13^C-xylose treatment, the SSU rRNA gene composition varied between days 1, 3, and 7 in corresponding high density fractions indicating different patterns of isotope incorporation into DNA on these days (Figure 2). In the ^13^C-xylose treatment on days 14 and 30 the SSU gene composition was similar to control on days 14 and 30 for corresponding high density fractions (Figure 2) indicating that ^13^C was no longer detectable in bacterial DNA on these days for this treatment. These results show that the dynamics of isotope incorporation into DNA varied considerably for organisms that assimilated C from either xylose or cellulose.

### Temporal dynamics of OTU relative abundance in non-fractionated DNA from soil

We monitored the soil microbial community over the course of the experiment by surveying SSU rRNA genes in non-fractionated DNA from the soil. The SSU rRNA gene composition of the non-fractionated DNA changed with time (Figure S4, *P* = 0.023, *R*^2^ = 0.63, permutational MANOVA with model formula:weighted unifrac distances ~ day as an ordered factor). In contrast, the microbial community could not be shown to change with treatment (*P*-value 0.23, permutational MANOVA with model formula:weighted unfrac distance ~ treatment; Figure S4). The latter result demonstrates that substitution of ^13^C-labeled substrates for unlabeled equivalents could not be shown to alter the soil microbial community composition. Twenty-nine OTUs exhibited sufficient statistical evidence (adjusted *P* < 0.10, Wald test) to conclude they changed in relative abundance in the non-fractionated DNA over time (Figure S5). When SSU rRNA gene abundances were combined at the taxonomic rank of “class,” the classes that changed in abundance (adjusted *P* < 0.10, Wald test) were the *Bacilli* (decreased), *Flavobacteria* (decreased), *Gammaproteobacteria* (decreased), and *Herpetosiphonales* (increased) (Figure S6). Of the 29 OTUs that changed in relative abundance over time, 14 putatively incorporated ^13^C into DNA (see below and Figure S5). OTUs that likely assimilated ^13^C from ^13^C-cellulose tended to increase in relative abundance with time whereas OTUs that assimilated ^13^C from ^13^C-xylose tended to decrease (Figure S5). OTUs that responded to both substrates did not exhibit a consistent relative abundance response over time (Figure S5).

### Changes in the phylogenetic composition of ^13^C-labeled OTUs with substrate and time

**Figure 3:**
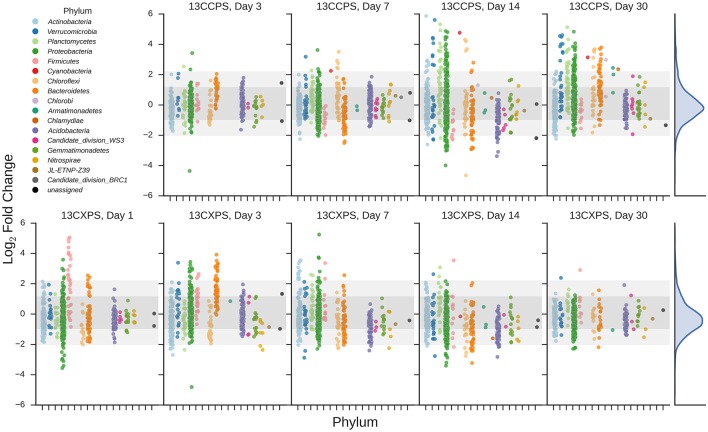
**Enrichment of OTUs in either ^13^C-cellulose (13CCPS, upper panels) or ^13^C-xylose (13CXPS, bottom panels) treatments relative to control, expressed as LFC (see Methods)**. Each point indicates the LFC for a single OTU. High enrichment values indicate an OTU is likely ^13^C-labeled. Different colors represent different phyla and different panels represent different days. The final column shows the frequency distribution of LFC values in each row. Within each panel, shaded areas are used to indicate one standard deviation (dark shading) or two standard deviations (light shading) about the mean of all LFC values.

**Figure 4:**
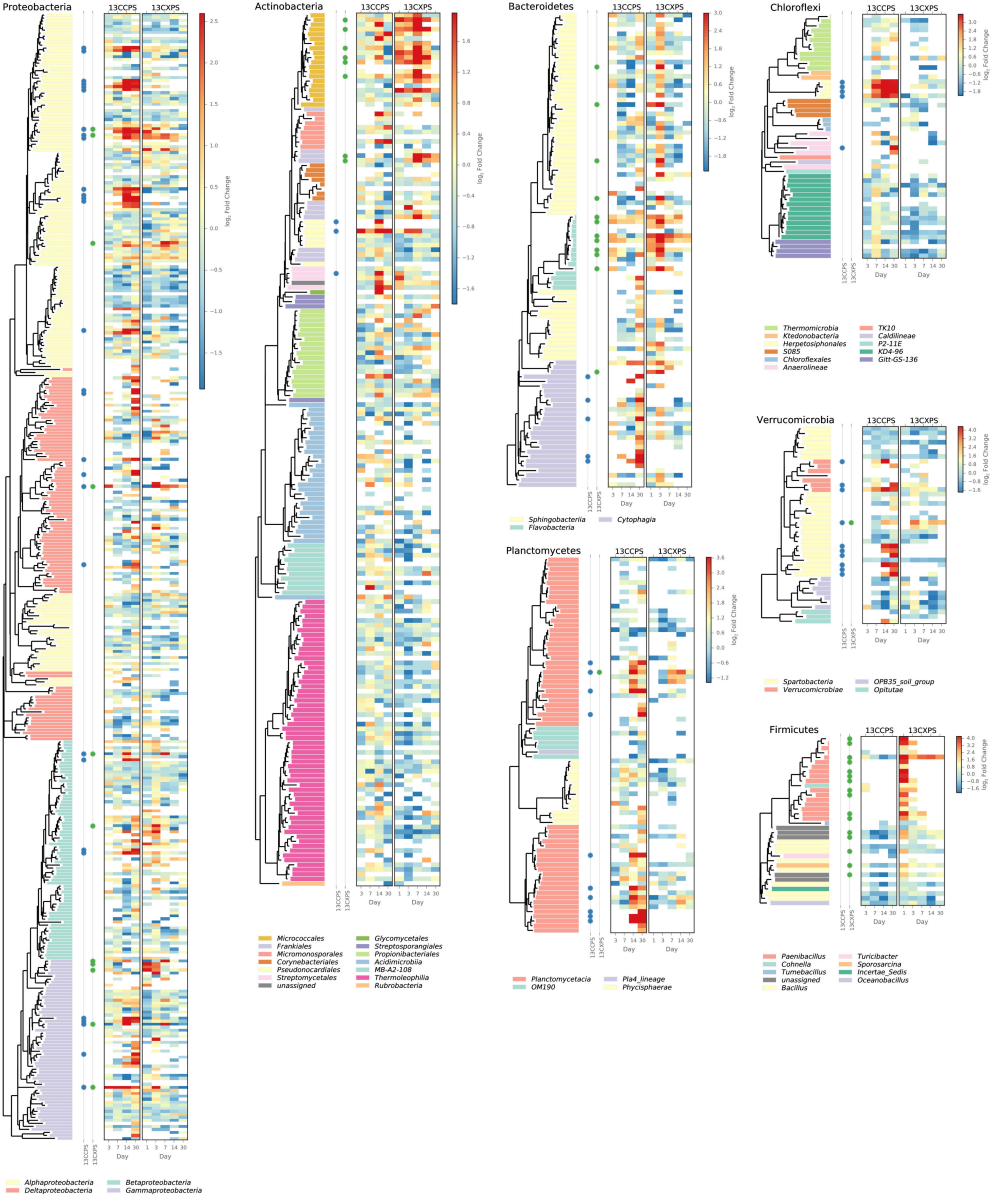
**Phylogenetic position of cellulose responders and xylose responders in the context of all OTUs that passed sparsity independent filtering criteria (see Methods)**. Only those phyla that contain responders are shown. Colored dots are used to identify xylose responders (green) and cellulose responders (blue). The heatmaps indicate enrichment in high density fractions relative to control (represented as LFC) for each OTU in response to both ^13^C-cellulose (13CCPS, leftmost heatmap) and ^13^C-xylose (13CXPS, rightmost heatmap) with values for different days in each heatmap column. High enrichment values (represented as LFC) provide evidence of ^13^C-labeled DNA.

If an OTU exhibited strong evidence for assimilating ^13^C into DNA, we refer to that OTU as a “responder” (see Methods for our operational definition of “responder”). The SSU rRNA gene sequences produced in this study were binned into 5940 OTUs and we assessed evidence of ^13^C-labeling from both ^13^C-cellulose and ^13^C-xylose for each OTU. Forty-one OTUs responded to ^13^C-xylose, 55 OTUs responded to ^13^C-cellulose, and 8 OTUs responded to both xylose and cellulose (Figures 3, 4, Figure S7, Tables S1, S2). The number of xylose responders peaked at days 1 and 3 and declined with time (Figure 1). In contrast, the number of cellulose responders increased with time peaking at days 14 and 30 (Figure 1).

**Figure 5:**
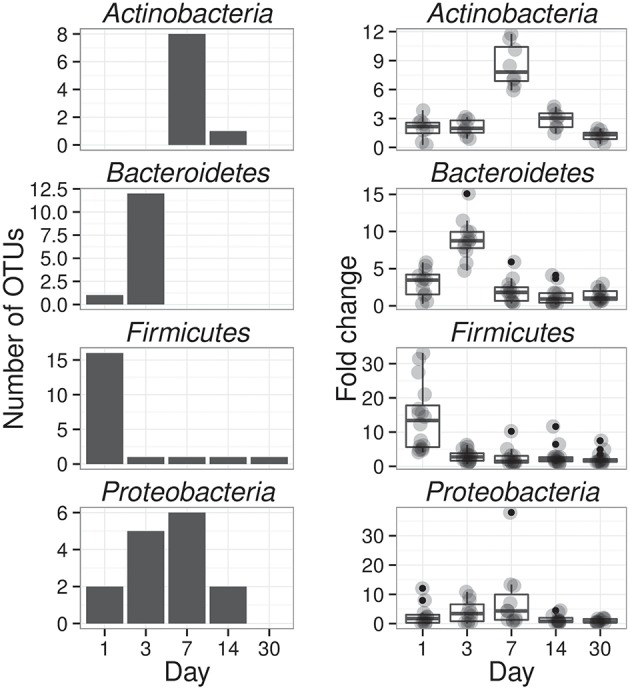
**Xylose reponders in the *Actinobacteria*, *Bacteroidetes*, *Firmicutes* exhibit distinct temporal dynamics of ^13^C-labeling**. The left column shows counts of ^13^C-xylose responders in the *Actinobacteria, Bacteroidetes, Firmicutes* and *Proteobacteria* at days 1, 3, 7, and 30. The right panel shows OTU enrichment in high density gradient fractions (gray points, expressed as fold change) for responders as well as a boxplot for the distribution of fold change values [The box extends one interquartile range, whiskers extend 1.5 times the IR, and small dots are outliers (i.e., beyond 1.5 times the IR)]. Each day in the right column shows all responders (i.e., OTUs that responded to xylose at any point in time). High enrichment values indicates OTU DNA is likely ^13^C-labeled.

**Figure 6:**
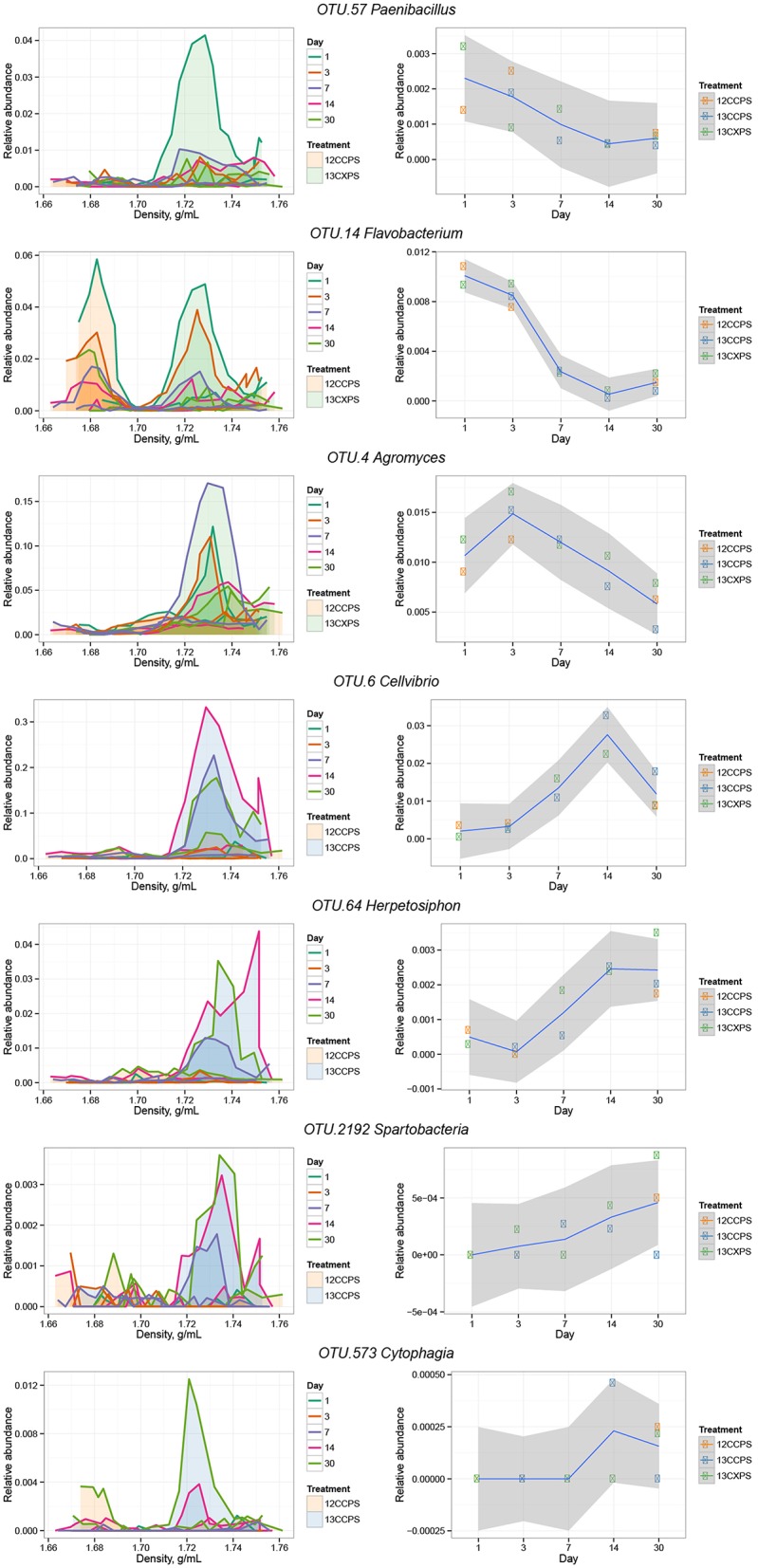
**Raw data from individual responders highlighted in the main text (see Results)**. The left column shows OTU relative abundance in density gradient fractions for the indicated treatment pair at each sampling point. Time is indicated by the line color (see legend). Gradient profiles are shaded to represent the different treatments where orange represents “control,” blue “^13^C-cellulose,” and green “^13^C-xylose.” The right column shows the relative abundance of each OTU in non-fractionated DNA. Enrichment in the high density fractions of ^13^C-treatments indicates an OTU likely has ^13^C-labeled DNA.

The phylogenetic composition of xylose responders changed with time (Figures 3, 5) and most xylose responders shared >97% SSU rRNA gene sequence identity with bacteria cultured in isolation (Table S1). On day 1, *Bacilli* OTUs represented 84% of xylose responders (Figure 5) and the majority of these OTUs were closely related to cultured representatives of the genus *Paenibacillus* (Table S1, Figure 4). For example, “OTU.57” had a strong signal of ^13^C-labeling at day 1 coinciding with its maximum relative abundance in non-fractionated DNA. The relative abundance of “OTU.57” declined until day 14 and it did not appear to be ^13^C-labeled after day 1 (Figure 6). On day 3, *Bacteroidetes* OTUs comprised 63% of xylose responders (Figure 5) and these OTUs were closely related to cultured representatives of the *Flavobacteriales* and *Sphingobacteriales* (Table S1, Figure 4). For example, “OTU.14,” annotated as a flavobacterium, had a strong signal for ^13^C-labeling at days 1 and 3 coinciding with its maximum relative abundance in non-fractionated DNA. Its relative abundance then declined until day 14 and did not show evidence of ^13^C-labeling beyond day 3 (Figure 6). Finally, on day 7, *Actinobacteria* OTUs represented 53% of the xylose responders (Figure 5) and these OTUs were closely related to cultured representatives of *Micrococcales* (Table S1, Figure 4). For example, “OTU.4,” annotated as *Agromyces*, had signal for ^13^C-labeling on days 1, 3 and 7 with the strongest evidence of ^13^C-labeling at day 7 and did not appear ^13^C-labeled at days 14 and 30. The relative abundance of “OTU.4” in non-fractionated DNA increased until day 3 and then declined until day 30 (Figure 6). *Proteobacteria* were also common among xylose responders at day 7 where they comprised 40% of xylose responder OTUs. Notably, *Proteobacteria* represented the majority (6 of 8) of OTUs that responded to both cellulose and xylose (Figure S7).

**Figure 7:**
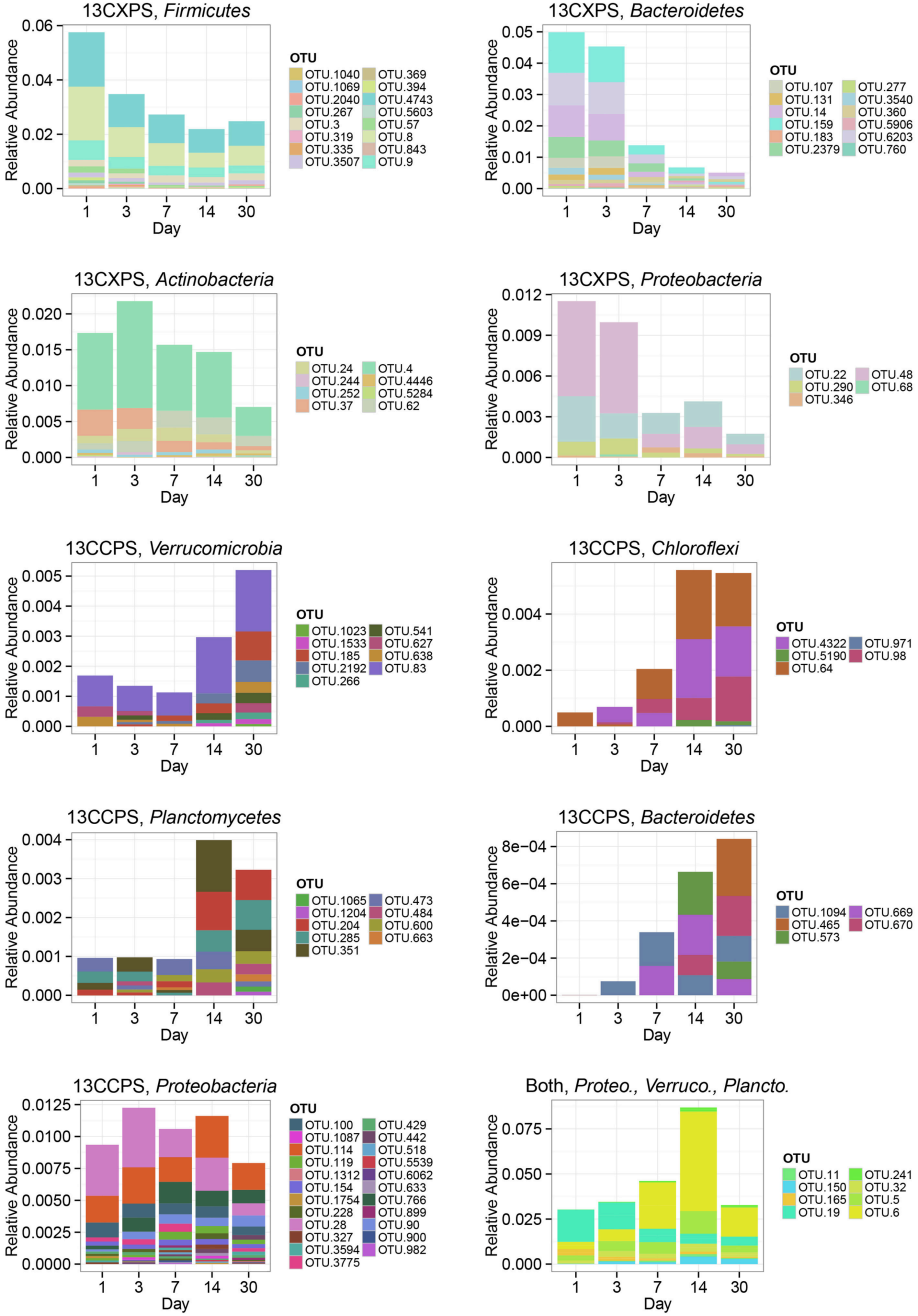
**Change in relative abundance in non-fractionated DNA over time for xylose responders (13CXPS) and cellulose responders (13CCPS)**. Each panel represents a responders to the indicated substrate [i.e., cellulose (13CCPS) or xylose (13CXPS)] within the indicated phylum except for the lower right panel which shows all reponders to both xylose and celluose. The abbreviations Proteo., Verruco., and Plancto., correspond to *Proteobacteria, Verrucomicrobia*, and *Planctomycetes*, respectively.

The phylogenetic composition of cellulose responders did not change with time to the same extent as the xylose responders. Both the relative abundance and the number of cellulose responders increased gradually over time peaking at days 14 and 30 (Figures 1, 3, 7). Cellulose responders belonged to the *Proteobacteria* (46%), *Verrucomicrobia* (16%), *Planctomycetes* (16%), *Chloroflexi* (8%), *Bacteroidetes* (8%), *Actinobacteria* (3%), and *Melainabacteria* (1 OTU; Table S2). Most cellulose responders increased in non-fractionated DNA relative abundance with time and had the strongest evidence for ^13^C-labeling at days 14 and 30 (e.g., “OTU.6,” “OTU.64,” “OTU.2192,” “OTU.573”; Figure 6). Also, in contrast to xylose responders, many cellulose responders were only distantly related to cultured isolates (Figure 4). For example, most of the *Verrucomicrobia* cellulose responders fell within unidentified *Spartobacteria* clades that had < 85% SSU rRNA identity to cultivated isolates (Figure 4). Likewise, *Chloroflexi* cellulose responders belonged to an unidentified clade within the *Herpetosiphonales* (Figure 4) and they shared < 89% SSU rRNA identity to cultivated isolates (Figure 4). *Bacteroidetes* included responders to both cellulose and xylose, though the former were members of *Cytophagales* while the latter belonged either to *Flavobacteriales* or *Sphingobacteriales* (Figure 4). Proteobacterial cellulose responders belonged to *Alpha* (13 OTUs), *Beta* (4 OTUs), *Gamma* (5 OTUs), and *Delta-proteobacteria* (6 OTUs). Most proteobacterial cellulose responders were closely related (>97% identity) to isolated strains, including representatives of the genera: *Cellvibrio, Devosia, Rhizobium*, and *Sorangium*, which are all known for their ability to degrade cellulose (Table S2).

### Characteristics of cellulose and xylose responders

**Figure 8:**
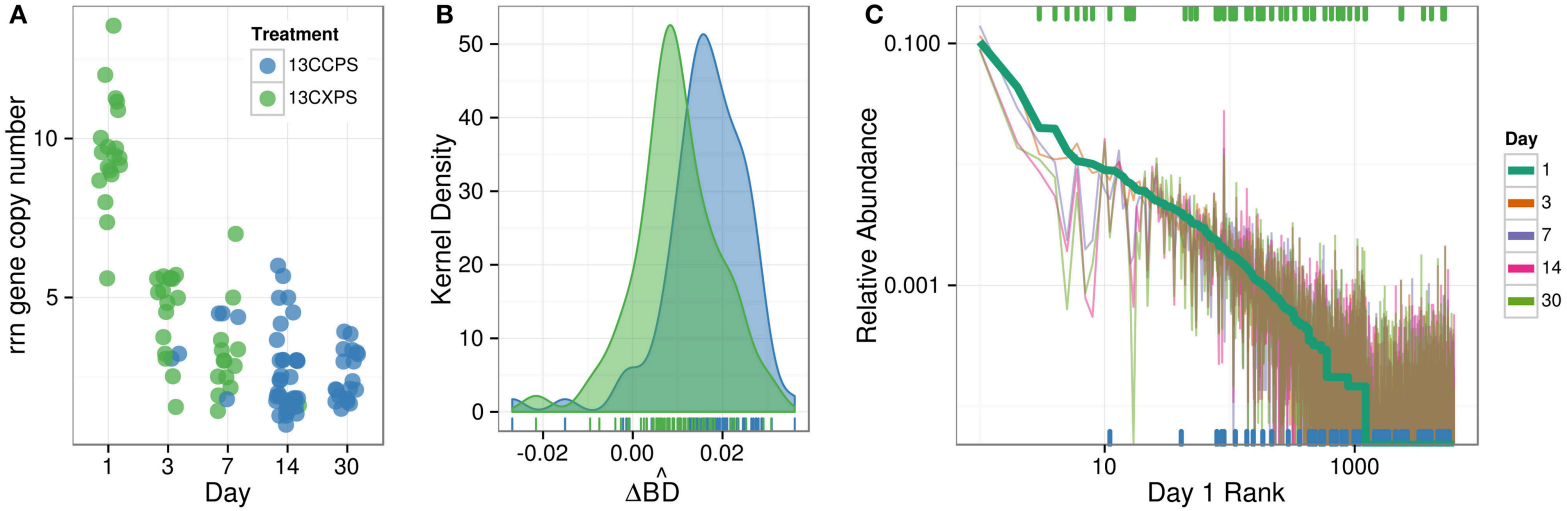
**Characteristics of xylose responders (green) and cellulose responders (blue) based on estimated *rrn* copy number (A), $\Delta \hat{BD}$ (B), and relative abundance in non-fractionated DNA (C)**. The estimated *rrn* copy number of all responders is shown vs. time **(A)**. Kernel density histogram of $\Delta \hat{BD}$ values shows cellulose responders had higher average $\Delta \hat{BD}$ than xylose responders indicating higher average atom % ^13^C in OTU DNA **(B)**. The final panel indicates the rank relative abundance of all OTUs observed in the non-fractionated DNA **(C)** where rank was determined at day 1 (bold line) and relative abundance for each OTU is indicated for all days by colored lines (see legend). Xylose responders (green ticks) have higher relative abundance in non-fractionated DNA than cellulose responders (blue ticks). All ticks are based on day 1 relative abundance.

Cellulose responders, relative to xylose responders, tended to have lower relative abundance in non-fractionated DNA, demonstrated signal consistent with higher atom % ^13^C in labeled DNA, and had lower estimated *rrn* copy number (Figure 8). OTUs that assimilated C from either cellulose or xylose were also clustered phylogenetically (see below) indicating that these traits were not dispersed randomly across bacterial species.

In the non-fractionated DNA, xylose responders [3.5 × 10^−3^ (s.d. 5.2 × 10^−3^)] had higher relative abundance than cellulose responders [1.2 × 10^−3^ (s.d. 3.8 × 10^−3^)] (Figure 8, *P* = 1.12 × 10^−5^, Wilcoxon Rank Sum test). Consistent with this observation, seven of the ten most abundant responders were xylose responders, and six of the ten most abundant OTUs overall (i.e., responders + non-responders) were xylose responders.

DNA buoyant density (BD) increases in proportion to atom % ^13^C. Hence, the extent of ^13^C incorporation into DNA can be evaluated by the difference in BD between ^13^C-labeled and unlabeled DNA. We calculated for each OTU its mean BD weighted by relative abundance to determine its “center of mass” within a given density gradient. We then quantified for each OTU the difference in center of mass between control gradients and gradients from ^13^C-xylose or ^13^C-cellulose treatments (see methods for the detailed calculation; Figure S2). We refer to the change in center of mass position for an OTU in response to ^13^C-labeling as $\Delta \hat{BD}$. Cellulose responder $\Delta \hat{BD}$ [0.0163 g mL^−1^ (s.d. 0.0094)] was greater than that of xylose responders (0.0097 g mL^−1^ (s.d. 0.0094); Figure 8, *P* = 1.8610 × 10^−6^, Wilcoxon Rank Sum test).

We predicted the *rrn* gene copy number for responders as described (Kembel et al., 2012). The ability to proliferate after rapid nutrient influx correlates positively to a microorganism's *rrn* copy number (Klappenbach et al., 2000). Cellulose responders possessed fewer estimated *rrn* copy numbers [2.7 (1.2 s.d.)] than xylose responders [6.2 (3.4 s.d.)] (*P* = 1.878 × 10^−9^, Wilcoxon Rank Sum test, Figure 8, Figure S8). Furthermore, the estimated *rrn* gene copy number for xylose responders was inversely related to the day of first response (*P* = 2.02 × 10^−15^, Wilcoxon Rank Sum test, Figure 8, Figure S8).

We assessed the phylogenetic clustering of ^13^C-responsive OTUs with NTI and NRI and quantified the average clade depth of cellulose and xylose responders with the consenTRAIT metric as described in methods. NRI values indicate that cellulose responders clustered overall and at the tips of the phylogeny (NRI: 4.49, NTI: 1.43) while xylose responders clustered terminally (NRI: −1.33, NTI: 2.69). The consenTRAIT clade depth for xylose and cellulose responders was 0.012 and 0.028 SSU rRNA gene sequence dissimilarity, respectively. As reference, the average clade depth as inferred from genomic analyses or growth in culture is ~0.017, 0.013, and 0.034 SSU rRNA gene sequence dissimilarity for arabinase (arabinose like xylose is a five C sugar found in hemicellulose), glucosidase and cellulase, respectively (Berlemont and Martiny, 2013; Martiny et al., 2013). These results indicate xylose responders form terminal clusters dispersed throughout the phylogeny while cellulose responders form deep clades of terminally clustered OTUs.

## Discussion

We highlight two key results with implications for understanding structure-function relationships in soils, and for applying DNA-SIP in future studies of the soil-C cycle. First, most cellulose responders belonged to undescribed taxonomic groups. This suggests we still have much to learn about the diversity of cellulolytic bacteria in soil. Second, the xylose response was characterized by a succession in activity from *Paenibacillus* OTUs (day 1) to *Bacteroidetes* (day 3) and finally *Micrococcales* (day 7). Notably, *Paenibacillus* have been previously shown by DNA-SIP to metabolize glucose (Verastegui et al., 2014). This activity succession was mirrored by relative abundance profiles and may indicate trophic-C exchange between these groups. While trophic exchange has been observed previously in DNA-SIP studies (Lueders et al., 2004b) most applications of DNA-SIP focus on proximal use of labeled substrates. However, with increased sensitivity, DNA-SIP is well-suited to tracking C flows throughout microbial communities over time. Though bacteria are often considered as a single trophic level (Moore et al., 1988), trophic interactions will critically influence how the global soil-C reservoir will respond to climate change (Crowther et al., 2015). Additionally, our results show that DNA-SIP results can change dramatically over time suggesting that multiple time points are necessary to rigorously and comprehensively describe which microorganisms consume ^13^C-labeled substrates in nucleic acid SIP incubations.

Bacteria that consumed ^13^C-cellulose were members of groups found widely in soil including *Spartobacteria, Planctomycetes*, and *Chloroflexi*, for which few related isolates are available in cultivation. Often cellulose responders were less than 90% related to their closest cultured relatives showing that we can infer little of their physiology from culture-based studies. *Verrucomicrobia* represented 16% of the cellulose responders. *Verrucomicrobia* are cosmopolitan soil microorganisms that can make up to 23% of SSU rRNA gene sequences in soils (Bergmann et al., 2011) and 9.8% of soil SSU rRNA (Buckley and Schmidt, 2001). Genomic analyses and laboratory experiments show that various isolates within the *Verrucomicrobia* are capable of methanotrophy, diazotrophy, and cellulose degradation (Wertz et al., 2011; Otsuka et al., 2012). Moreover, *Verrucomicrobia* have been hypothesized to degrade polysaccharides in many environments (Chin et al., 1999; Fierer et al., 2013; Herlemann et al., 2013). The majority of verrucomicrobial cellulose responders belonged to two clades that fell within the *Spartobacteria* (Figure 4). *Spartobacteria* outnumbered all other *Verrucomicrobia* phylotypes in SSU rRNA gene surveys of 181 globally distributed soil samples (Bergmann et al., 2011). Given their ubiquity and abundance in soil as well as their demonstrated incorporation of ^13^C from ^13^C-cellulose, *Verrucomicrobia* lineages, particularly *Spartobacteria*, may be important contributors to global cellulose turnover.

Other notable cellulose responders include OTUs in the *Planctomycetes* and *Chloroflexi* both of which have previously been shown to assimilate ^13^C from ^13^C-cellulose added to soil (Schellenberger et al., 2010). *Planctomycetes* are common in soil (Janssen, 2006), comprising 4 to 7% of bacterial cells in many soils (Zarda et al., 1997; Chatzinotas et al., 1998) and 7 ± 5% of SSU rRNA (Buckley and Schmidt, 2003). Although soil *Planctomycetes* are widespread, their activities in soil remain uncharacterized. *Plantomycetes* represented 16% of cellulose responders and shared <92% SSU rRNA gene sequence identity to their most closely related cultured isolates. *Chloroflexi* are known for metabolically diverse lifestyles ranging from anoxygenic phototrophy to organohalide respiration (Hug et al., 2013) and are among the six most abundant bacterial phyla in soil (Janssen, 2006). Recent studies have focused on *Chloroflexi* roles in C-cycling (Goldfarb et al., 2011; Cole et al., 2013; Hug et al., 2013) and several *Chloroflexi* isolates use cellulose (Goldfarb et al., 2011; Cole et al., 2013; Hug et al., 2013). Four of the five *Chloroflexi* cellulose responders belong to a single clade within the *Herpetosiphonales* (Figure 4).

Finally, a single cellulose responder belonged to the *Melainabacteria* phylum (95% shared SSU rRNA gene sequence identity with *Vampirovibrio chlorellavorus*). The phylogenetic position of *Melainabacteria* is debated but *Melainabacteria* have been proposed to be a non-phototrophic sister phylum to *Cyanobacteria*. An analysis of a *Melainabacteria* genome (Rienzi et al., 2013) suggests the genomic capacity to degrade polysaccharides though *Vampirovibrio chlorellavorus* is an obligate predator of green alga (Gromov and Mamkaeva, 1972).

The turnover of cellulose and plant-derived sugars in soil has been studied previously using DNA-SIP (e.g., Verastegui et al., 2014). Similar to our study, phylotypes among the *Chloroflexi, Bacteroidetes*, and *Planctomycetes* have all been previously implicated in soil cellulose degradation (Schellenberger et al., 2010). Additionally, functional metagenomics enabled by DNA-SIP has identified glycoside hydrolases putatively belonging to *Cellvibrio* and *Spartobacteria* further suggesting a role for these organisms in cellulose breakdown in soils (Verastegui et al., 2014). Fungi undoubtedly also contribute to the decomposition of cellulose in soils (Boer et al., 2005), but they are not a focus of this experiment.

In addition to taxonomic identity, we quantified four ecological properties of microorganisms that were actively engaged in labile and structural C decomposition in our experiment: (1) time of activity, (2) estimated *rrn* gene copy number, (3) phylogenetic clustering, and (4) density shift in response to ^13^C-labeling. Xylose was consumed before cellulose and these substrates were consumed by different microorganisms (Figures 1–3). Consumers of xylose had higher estimated *rrn* gene copy number than cellulose consumers (Figure 8). *rrn* copy number is positively correlated with the ability to resuscitate quickly in response to nutrient influx (Klappenbach et al., 2000) which may be the advantage that enabled xylose responders to rapidly consume xylose. Both xylose and cellulose responders were terminally clustered in phylogenetic trees suggesting that the ability to use these substrates was phylogenetically constrained. Although labile C consumption is generally considered to be mediated by a diverse set of microorganisms, we found that xylose responders at day 1 were mainly members of one genus, *Paenibacillus* (Figure 4). Our results suggest that life-history traits such as the ability to resuscitate quickly and/or grow rapidly may be more important in determining the diversity of microorganisms that actually mediate a given process than the genomic potential for substrate utilization. And last, xylose consumers, in contrast to cellulose consumers, had lower $\Delta \hat{BD}$ in response to ^13^C-labeling. This result suggests that many xylose C consumers were generalists, assimilating C from a variety of sources both labeled and unlabeled, while structural C consumers were more likely to be specialists and more closely associated with C from a single source.

Responders do not necessarily assimilate ^13^C directly from ^13^C-xylose or ^13^C-cellulose, and in many ways, knowledge of secondary C-transformations and/or microbial biomass turnover may be more interesting with respect to the soil C-cycle than knowledge of primary degradation. In the case of cellulose degradation, non-cellulolytic bacteria may gain access to ^13^C from ^13^C-cellulose via ecological interactions including mutualism, parasitism, predation, and ‘cheating’. Cheaters in particular, who benefit from the activities of cellulolytic extracellular enzymes without bearing the costs of producing these enzymes, have been hypothesized to impact rates of cellulose degradation (Allison, 2005). Cheaters should be able to use oligosaccharides and sugars produced by extracellular enzymes. We note with interest that few OTUs responded both to ^13^C-cellulose and ^13^C-xylose. This means that if these responders include taxa labeled by secondary C-transformations, then these secondary C-transformations are substrate specific.

The response to ^13^C -xylose suggests that xylose-C moved through different trophic levels within the soil bacterial food web. Trophic exchange would explain well the precipitous drop in abundance of *Paenibacillus* after day 1 with subsequent ^13^C-labeling of *Bacteroidetes* at day 3 as well as the precipitous drop in abundance of *Bacteroidetes* at day 3 followed by ^13^C-labeling of *Micrococcales* at day 7. The *Bacilli* degraded xylose first (65% of the xylose-C had been respired by day 1) representing 84% of day 1 xylose responders (Figure 5). *Bacilli* also comprised about 6% of SSU rRNA genes present in non-fractionated DNA on day 1 (Figure 7). However, few *Bacilli* remained ^13^C-labeled by day 3 and their abundance declined reaching about 2% of soil SSU rRNA genes by day 30 (Figures 5, 7). Members of the *Bacillus* (Cleveland et al., 2007) and *Paenibacillus* in particular (Verastegui et al., 2014) have been previously implicated as labile C decomposers. *Bacteroidetes* OTUs appeared ^13^C-labeled at day 3 concomitant with the decline in relative abundance and loss of ^13^C-label for *Bacilli*. Finally, *Actinobacteria* appeared ^13^C-labeled at day 7 as *Bacteroidetes* xylose responders declined in relative abundance and became unlabeled. Hence, it seems reasonable to propose that *Bacteroidetes* and *Actinobacteria* xylose responders became labeled via the consumption of ^13^C derived from ^13^C-labeled microbial biomass as opposed to primary degradation of ^13^C-xylose. Trophic exchange could be enabled by mother cell lysis (in the case of spore formers such as *Paenibacillus*), viral lysis, and/or predation.

The inferred physiology of *Actinobacteria* and *Bacteroidetes* xylose responders provides further evidence for C transfer by saprotrophy and/or predation. Certain *Bacteroidetes* have been shown to become ^13^C-labeled after the addition of live ^13^C-labeled *Escherichia coli* to soil (Lueders et al., 2006) indicating their ability to assimilate C from microbial biomass. In addition, the dominant OTU labeled in the ^13^C-xylose treatment from the *Micrococcales* shares 100% SSU rRNA gene sequence identity to *Agromyces ramosus* a known predator that feeds upon on many microorganisms including yeast and *Micrococcus luteus* (Casida, 1983). *Agromyces* are abundant microorganisms in many soils and *Agromyces ramosus* was the most abundant xylose responder in our experiment—and the fourth most abundant OTU in our dataset. It is notable however, that *Agromyces ramosus* remains unlabeled in response to ^13^C-cellulose. This result suggests that if *Agromyces ramosus* is acting as a predator in our experiment, it is either a selective predator or it is only active under a restricted set of certain environmental conditions.

If trophic transfer caused the dynamics we observed, at least three different ecological groups exchanged C in 7 days. Models of the soil C cycle often exclude trophic interactions between soil bacteria [e.g., Moore et al. (1988)], yet when soil C models do account for predators and/or saprophytes, trophic interactions are predicted to have significant effects on the fate of soil C (Kaiser et al., 2014). Climate change is expected to diminish bottom-up controls on microbial growth increasing the importance on top-down biological interactions for mitigating positive climate change feedbacks (Crowther et al., 2015). Currently the extent of bacterial predatory activity in soil, and its consequences for the soil C-cycle and carbon use efficiency is largely unknown. Elucidating the identities of bacterial predators in soil will assist in assessing the implications of climate change on global soil-C storage.

Microorganisms govern C-transformations in soil and thereby influence global climate but we still do not know the specific identities of microorganisms that carry out critical C transformations. In this experiment, cellulose responders included members of the *Verrucomicrobia* (*Spartobacteria*), *Chloroflexi, Bacteroidetes*, and *Planctomycetes*. *Spartobacteria* in particular are globally cosmopolitan soil microorganisms and are often the most abundant *Verrucomicrobia* in soil (Bergmann et al., 2011). Our results suggest three major conclusions: (i) as yet uncharacterized cellulolytic bacteria appear to be present in several major lineages of uncultivated bacteria found widely in soil, (ii) most of the OTUs that responded to xylose were labeled as a result of trophic transfer and not primary assimilation, and (iii) life history traits are likely to act as a filter constraining the diversity of active microorganisms relative to those with the genomic potential for a given metabolism.

## Author contributions

CP conceived, developed and implemented data analysis and interpreted data, AC designed and performed the experiments, and both CP and AC performed data analysis, generated figures, and wrote and edited the manuscript. CP and AC contributed equally to the manuscript and should be considered co-first authors. CK and SB assisted with sampling and experimental analyses including development and implementation of the DNA sequencing pipeline. DB conceived the experimental design and supervised all aspects of experimentation, data analysis, and manuscript preparation.

### Conflict of interest statement

The authors declare that the research was conducted in the absence of any commercial or financial relationships that could be construed as a potential conflict of interest.

## References

[B1] Allison S. D. (2005). Cheaters, diffusion and nutrients constrain decomposition by microbial enzymes in spatially structured environments. Ecol. Lett. 8, 626–635. 10.1111/j.1461-0248.2005.e6.x

[B2] Allison S. D. Wallenstein M. D. Bradford M. A. (2010). Soil-carbon response to warming dependent on microbial physiology. Nat. Geosci. 3, 336–340. 10.1038/ngeo846

[B3] Altschul S. F. Gish W. Miller W. Myers E. W. Lipman D. J. (1990). Basic local alignment search tool. J. Mol. Biol. 215, 403–410. 10.1016/S0022-2836(05)80360-2 2231712

[B4] Amundson R. (2001). The carbon budget in soils. Annu. Rev. Earth Planet. Sci. 29, 535–562. 10.1146/annurev.earth.29.1.535

[B5] Anderson M. J. (2001). A new method for non-parametric multivariate analysis of variance. Austral. Ecol. 26, 32–46. 10.1111/j.1442-9993.2001.01070.pp.x

[B6] Aoyagi T. Hanada S. Itoh H. Sato Y. Ogata A. Friedrich M. W.. (2015). Ultra-high-sensitivity stable-isotope probing of rRNA by high-throughput sequencing of isopycnic centrifugation gradients. Environ. Microbiol. Rep. 7, 282–287. 10.1111/1758-2229.12243 25403652

[B7] Batjes N. H. (1996). Total carbon and nitrogen in the soils of the world. Eur. J. Soil Sci. 47, 151–163. 10.1111/j.1365-2389.1996.tb01386.x

[B8] Benedict S. R. (1909). A reagent for the detection of reducing sugars. J. Biol. Chem. 5, 485–487. 11953443

[B9] Benjamini Y. Hochberg Y. (1995). Controlling the false discovery rate: a practical and powerful approach to multiple testing. J. R. Stat. Soc. Series B 57, 289–300.

[B10] Bergmann G. T. Bates S. T. Eilers K. G. Lauber C. L. Caporaso J. G. Walters W. A.. (2011). The under-recognized dominance of *Verrucomicrobia* in soil bacterial communities. Soil Biol. Biochem. 43, 1450–1455. 10.1016/j.soilbio.2011.03.012 22267877 PMC3260529

[B11] Berlemont R. Martiny A. C. (2013). Phylogenetic distribution of potential cellulases in bacteria. Appl. Environ. Microbiol. 79, 1545–1554. 10.1128/AEM.03305-12 23263967 PMC3591946

[B12] Berthrong S. T. Buckley D. H. Drinkwater L. E. (2013). Agricultural management and labile carbon additions affect soil microbial community structure and interact with carbon and nitrogen cycling. Microb. Ecol. 66, 158–170. 10.1007/s00248-013-0225-0 23588849

[B13] Birnie G. D. (1978). Centrifugal Separations in Molecular and Cell Biology. Boston, MA: Butterworth & Co Publishers Ltd.

[B14] Boer W. de Folman L. B. Summerbell R. C. Boddy L. (2005). Living in a fungal world: impact of fungi on soil bacterial niche development. FEMS Microbiol. Rev. 29, 795–811. 10.1016/j.femsre.2004.11.005 16102603

[B15] Bradford M. A. Fierer N. Reynolds J. F. (2008). Soil carbon stocks in experimental mesocosms are dependent on the rate of labile carbon, nitrogen and phosphorus inputs to soils. Funct. Ecol. 22, 964–974. 10.1111/j.1365-2435.2008.01404.x

[B16] Bradford M. M. (1976). A rapid and sensitive method for the quantitation of microgram quantities of protein utilizing the principle of protein-dye binding. Anal. Biochem. 72, 248–254. 10.1016/0003-2697(76)90527-3 942051

[B17] Buckley D. H. Huangyutitham V. Hsu S. F. Nelson T. A. (2007). Stable isotope probing with ^15^N achieved by disentangling the effects of genome G + C content and isotope enrichment on DNA density. Appl. Environ. Microbiol. 73, 3189–3195. 10.1128/AEM.02609-06 17369331 PMC1907112

[B18] Buckley D. H. Schmidt T. M. (2001). Environmental factors influencing the distribution of rRNA from *Verrucomicrobia* in soil. FEMS Microbiol. Ecol. 35, 105–112. 10.1111/j.1574-6941.2001.tb00793.x 11248395

[B19] Buckley D. H. Schmidt T. M. (2002). Exploring the diversity of soil - a microbial rainforest, in Biodiversity of Microbial Life: Foundation of Earths Biosphere, ed Reysenbach A. L. (New York, NY: Wiley), 183–208.

[B20] Buckley D. H. Schmidt T. M. (2003). Diversity and dynamics of microbial communities in soils from agro-ecosystems. Environ. Microbiol. 5, 441–452. 10.1046/j.1462-2920.2003.00404.x 12755711

[B21] Camacho C. Coulouris G. Avagyan V. Ma N. Papadopoulos J. Bealer K.. (2009). BLAST+: architecture and applications. BMC Bioinformatics 10:421. 10.1186/1471-2105-10-421 20003500 PMC2803857

[B22] Caporaso J. G. Kuczynski J. Stombaugh J. Bittinger K. Bushman F. D. Costello E. K.. (2010). QIIME allows analysis of high-throughput community sequencing data. Nat. Methods 7, 335–336. 10.1038/nmeth.f.303 20383131 PMC3156573

[B23] Casida L. E. (1983). Interaction of *Agromyces ramosus* with other bacteria in soil. Appl. Environ. Microbiol. 46, 881–888. 16346402 10.1128/aem.46.4.881-888.1983 PMC239483

[B24] Chapin F. (2002). Principles of Terrestrial Ecosystem Ecology. New York, NY: Springer.

[B25] Chatzinotas A. Sandaa R. A. Schönhuber W. Amann R. Daae F. L. Torsvik V.. (1998). Analysis of broad-scale differences in microbial community composition of two pristine forest soils. Syst. Appl. Microbiol. 21, 579–587. 10.1016/S0723-2020(98)80070-2 9924826

[B26] Chen Y. Murrell J. C. (2010). When metagenomics meets stable-isotope probing: progress and perspectives. Trends Microbiol. 18, 157–163. 10.1016/j.tim.2010.02.002 20202846

[B27] Chin K.-J. Hahn D. Hengstmann U. Liesack W. Janssen P. H. (1999). Characterization and identification of numerically abundant culturable bacteria from the anoxic bulk soil of rice paddy microcosms. Appl. Environ. Microbiol. 65, 5042–5049. 10543821 10.1128/aem.65.11.5042-5049.1999 PMC91679

[B28] Cleveland C. C. Nemergut D. R. Schmidt S. K. Townsend A. R. (2007). Increases in soil respiration following labile carbon additions linked to rapid shifts in soil microbial community composition. Biogeochemistry 82, 229–240. 10.1007/s10533-006-9065-z

[B29] Cole J. K. Gieler B. A. Heisler D. L. Palisoc M. M. Williams A. J. Dohnalkova A. C. (2013). *Kallotenue papyrolyticum* gen. nov. sp. nov., a cellulolytic and filamentous thermophile that represents a novel lineage (Kallotenuales ord. nov., Kallotenuaceae fam. nov.) within the class Chloroflexia. Int. J. Syst. Evol. Microbiol. 63, 4675–4682. 10.1099/ijs.0.053348-0 23950149

[B30] Coleman D. C. Crossley D. A. (1996). Fundamentals of Soil Ecology. Waltham, MA: Academic Press.

[B31] Crowther T. W. Thomas S. M. Maynard D. S. Baldrian P. Covey K. Frey S. D.. (2015). Biotic interactions mediate soil microbial feedbacks to climate change. Proc. Natl. Acad. Sci. U.S.A. 112, 7033–7038. 10.1073/pnas.1502956112 26038557 PMC4460469

[B32] Datta R. Vranová V. Pavelka M. Rejšek K. Formánek P. (2014). Effect of soil sieving on respiration induced by low-molecular-weight substrates. Int. Agrophys. 28, 119–124. 10.2478/intag-2013-0034

[B33] David K. Ragauskas A. J. (2010). Switchgrass as an energy crop for biofuel production: a review of its ligno-cellulosic chemical properties. Energy Environ. Sci. 3, 1182 10.1039/b926617h

[B34] DeRito C. M. Pumphrey G. M. Madsen E. L. (2005a). Use of field-based stable isotope probing to identify adapted populations and track carbon flow through a phenol-degrading soil microbial community. Appl. Environ. Microbiol. 71, 7858–7865. 10.1128/AEM.71.12.7858-7865.2005 16332760 PMC1317415

[B35] DeRito C. M. Pumphrey G. M. Madsen E. L. (2005b). Use of field-based stable isotope probing to identify adapted populations and track carbon flow through a phenol-degrading soil microbial community. Appl. Environ. Microbiol. 71, 7858–7865. 10.1128/AEM.71.12.7858-7865.2005 16332760 PMC1317415

[B36] DeSantis T. Z. Hugenholtz P. Keller K. Brodie E. L. Larsen N. Piceno Y. M.. (2006). NAST: a multiple sequence alignment server for comparative analysis of 16S rRNA genes. Nucleic Acids Res. 34, W394–W399. 10.1093/nar/gkl244 16845035 PMC1538769

[B37] Edgar R. C. (2010). Search and clustering orders of magnitude faster than BLAST. Bioinformatics 26, 2460–2461. 10.1093/bioinformatics/btq461 20709691

[B38] Edgar R. C. (2013). UPARSE: highly accurate OTU sequences from microbial amplicon reads. Nat. Methods 10, 996–998. 10.1038/nmeth.2604 23955772

[B39] Evans S. E. Wallenstein M. D. (2014). Climate change alters ecological strategies of soil bacteria. Ecol. Lett. 17, 155–164. 10.1111/ele.12206 24261594

[B40] Fierer N. Ladau J. Clemente J. C. Leff J. W. Owens S. M. Pollard K. S.. (2013). Reconstructing the microbial diversity and function of pre-agricultural tallgrass prairie soils in the united states. Science 342, 621–624. 10.1126/science.1243768 24179225

[B41] Goldfarb K. C. Karaoz U. Hanson C. A. Santee C. A. Bradford M. A. Treseder K. K.. (2011). Differential growth responses of soil bacterial taxa to carbon substrates of varying chemical recalcitrance. Front. Microbiol. 2:94. 10.3389/fmicb.2011.00094 21833332 PMC3153052

[B42] Griffiths R. I. Whiteley A. S. O'Donnell A. G. Bailey M. J. (2000). Rapid method for coextraction of DNA and RNA from natural environments for analysis of ribosomal DNA- and rRNA-based microbial community composition. Appl. Environ. Microbiol. 66, 5488–5491. 10.1128/aem.66.12.5488-5491.2000 11097934 PMC92488

[B43] Gromov B. V. Mamkaeva K. A. (1972). Electron microscopic study of parasitism by *Bdellovibrio chlorellavorus* bacteria on cells of the green alga *Chlorella vulgaris*. Tsitologiia 14, 256–260. 5011884

[B44] Heo M.-S. Son H.-J. (2002). Development of an optimized, simple chemically defined medium for bacterial cellulose production by *Acetobacter* sp. A9 in shaking cultures. Biotechnol. Appl. Biochem. 36, 41–45. 10.1042/BA20020018 12149121

[B45] Herlemann D. P. R. Lundin D. Labrenz M. Jurgens K. Zheng Z. Aspeborg H.. (2013). Metagenomic *de novo* assembly of an aquatic representative of the verrucomicrobial class *Spartobacteria*. MBio 4:e0056912. 10.1128/mBio.00569-12 23716574 PMC3663571

[B46] Hsu S.-F. Buckley D. H. (2009). Evidence for the functional significance of diazotroph community structure in soil. ISME J. 3, 124–136. 10.1038/ismej.2008.82 18769458

[B47] Hug L. A. Castelle C. J. Wrighton K. C. Thomas B. C. Sharon I. Frischkorn K. R.. (2013). Community genomic analyses constrain the distribution of metabolic traits across the *Chloroflexi* phylum and indicate roles in sediment carbon cycling. Microbiome 1:22. 10.1186/2049-2618-1-22 24450983 PMC3971608

[B48] Janssen P. H. (2006). Identifying the dominant soil bacterial taxa in libraries of 16S rRNA and 16S rRNA genes. Appl. Environ. Microbiol. 72, 1719–1728. 10.1128/AEM.72.3.1719-1728.2006 16517615 PMC1393246

[B49] Jombart T. Dray S. (2010). Adephylo: exploratory analyses for the phylogenetic comparative method. Bioinformatics 26, 1907–1909. 10.1093/bioinformatics/btq292 20525823

[B50] Kaiser C. Franklin O. Dieckmann U. Richter A. (2014). Microbial community dynamics alleviate stoichiometric constraints during litter decay. Ecol. Lett. 17, 680–690. 10.1111/ele.12269 24628731 PMC4315898

[B51] Kembel S. Cowan P. Helmus M. Cornwell W. Morlon H. Ackerly D.. (2010). Picante: R tools for integrating phylogenies and ecology. Bioinformatics 26, 1463–1464. 10.1093/bioinformatics/btq166 20395285

[B52] Kembel S. W. Wu M. Eisen J. A. Green J. L. (2012). Incorporating 16S gene copy number information improves estimates of microbial diversity and abundance. PLoS Comput. Biol. 8:e1002743. 10.1371/journal.pcbi.1002743 23133348 PMC3486904

[B53] Klappenbach J. A. Dunbar J. M. Schmidt T. M. (2000). rRNA Operon copy number reflects ecological strategies of bacteria. Appl. Environ. Microbiol. 66, 1328–1333. 10.1128/AEM.66.4.1328-1333.2000 10742207 PMC91988

[B54] Linn D. M. Doran J. W. (1984). Aerobic and anaerobic microbial populations in no-till and plowed soils. Soil Sci. Soc. Am. J. 48, 794 10.2136/sssaj1984.03615995004800040019x

[B55] Love M. I. Huber W. Anders S. (2014). Moderated estimation of fold change and dispersion for RNA-seq data with DESeq2. Genome Biol. 15:550. 10.1186/s13059-014-0550-8 25516281 PMC4302049

[B56] Lozupone C. Knight R. (2005). UniFrac: a new phylogenetic method for comparing microbial communities. Appl. Environ. Microbiol. 71, 8228–8235. 10.1128/AEM.71.12.8228-8235.2005 16332807 PMC1317376

[B57] Lueders T. Kindler R. Miltner A. Friedrich M. W. Kaestner M. (2006). Identification of bacterial micropredators distinctively active in a soil microbial food web. Appl. Environ. Microbiol. 72, 5342–5348. 10.1128/AEM.00400-06 16885285 PMC1538704

[B58] Lueders T. Pommerenke B. Friedrich M. W. (2004a). Stable-isotope probing of microorganisms thriving at thermodynamic limits: syntrophic propionate oxidation in flooded soil. Appl. Environ. Microbiol. 70, 5778–5786. 10.1128/AEM.70.10.5778-5786.2004 15466514 PMC522077

[B59] Lueders T. Wagner B. Claus P. Friedrich M. W. (2004b). Stable isotope probing of rRNA and DNA reveals a dynamic methylotroph community and trophic interactions with fungi and protozoa in oxic rice field soil. Environ. Microbiol. 6, 60–72. 10.1046/j.1462-2920.2003.00535.x 14686942

[B60] Manefield M. Whiteley A. S. Griffiths R. I. Bailey M. J. (2002). RNA Stable isotope probing a novel means of linking microbial community function to phylogeny. Appl. Environ. Microbiol. 68, 5367–5373. 10.1128/AEM.68.11.5367-5373.2002 12406726 PMC129944

[B61] Martiny A. C. Treseder K. Pusch G. (2013). Phylogenetic conservatism of functional traits in microorganisms. ISME J. 7, 830–838. 10.1038/ismej.2012.160 23235290 PMC3603392

[B62] Matsen F. A. Kodner R. B. Armbrust E. V. (2010). Pplacer: Linear time maximum-likelihood and bayesian phylogenetic placement of sequences onto a fixed reference tree. BMC Bioinformatics 11:538. 10.1186/1471-2105-11-538 21034504 PMC3098090

[B63] McGuire K. L. Treseder K. K. (2010). Microbial communities and their relevance for ecosystem models: decomposition as a case study. Soil Biol. Biochem. 42, 529–535. 10.1016/j.soilbio.2009.11.016

[B64] McMurdie P. J. Holmes S. (2013). Phyloseq: an r package for reproducible interactive analysis and graphics of microbiome census data. PLoS ONE 8:e61217. 10.1371/journal.pone.0061217 23630581 PMC3632530

[B65] McMurdie P. J. Holmes S. (2014). Waste not, want not: why rarefying microbiome data is inadmissible. PLoS Comput. Biol. 10:e1003531. 10.1371/journal.pcbi.1003531 24699258 PMC3974642

[B66] Moore J. C. Walter D. E. Hunt H. W. (1988). Arthropod regulation of micro- and mesobiota in below-ground detrital food webs. Annu. Rev. Entomol. 33, 419–435. 10.1146/annurev.en.33.010188.002223

[B67] Nannipieri P. Ascher J. Ceccherini M. T. Landi L. Pietramellara G. Renella G. (2003). Microbial diversity and soil functions. Eur. J. Soil Sci. 54, 655–670. 10.1046/j.1351-0754.2003.0556.x

[B68] Nawrocki E. P. Eddy S. R. (2013). Infernal 1.1: 100-fold faster RNA homology searches. Bioinformatics 29, 2933–2935. 10.1093/bioinformatics/btt509 24008419 PMC3810854

[B69] Nawrocki E. P. Kolbe D. L. Eddy S. R. (2009). Infernal 1.0: inference of RNA alignments. Bioinformatics 25, 1335–1337. 10.1093/bioinformatics/btp157 19307242 PMC2732312

[B70] Neff J. C. Asner G. P. (2001). Dissolved organic carbon in terrestrial ecosystems: synthesis and a model. Ecosystems 4, 29–48. 10.1007/s100210000058

[B71] Oksanen J. Blanchet F. G. Kindt R. Legendre P. Minchin P. R. O'Hara R. B. (2015). Vegan: Community Ecology Package. Available online at: http://CRAN.R-project.org/package=vegan

[B72] Otsuka S. Ueda H. Suenaga T. Uchino Y. Hamada M. Yokota A. (2012). *Roseimicrobium gellanilyticum* gen. nov. sp. nov., a new member of the class *Verrucomicrobiae*. Int. J. Syst. Evol. Microbiol. 63, 1982–1986. 10.1099/ijs.0.041848-0 23041636

[B73] Price M. N. Dehal P. S. Arkin A. P. (2010). FastTree2 - approximately maximum-likelihood trees for large alignments. PLoS ONE 5:e9490. 10.1371/journal.pone.0009490 20224823 PMC2835736

[B74] Quast C. Pruesse E. Yilmaz P. Gerken J. Schweer T. Yarza P.. (2013). The SILVA ribosomal RNA gene database project: improved data processing and web-based tools. Nucleic Acids Res. 41, D590–D596. 10.1093/nar/gks1219 23193283 PMC3531112

[B75] Radajewski S. Ineson P. Parekh N. R. Murrell J. C. (2000). Stable-isotope probing as a tool in microbial ecology. Nature 403, 646–649. 10.1038/35001054 10688198

[B76] Reed H. E. Martiny J. B. H. (2007). Testing the functional significance of microbial composition in natural communities. FEMS Microbiol. Ecol. 62, 161–170. 10.1111/j.1574-6941.2007.00386.x 17937673

[B77] Rienzi S. C. D. Sharon I. Wrighton K. C. Koren O. Hug L. A. Thomas B. C.. (2013). The human gut and groundwater harbor non-photosynthetic bacteria belonging to a new candidate phylum sibling to *Cyanobacteria*. Elife 2:e01102. 10.7554/elife.01102 24137540 PMC3787301

[B78] Schellenberger S. Kolb S. Drake H. L. (2010). Metabolic responses of novel cellulolytic and saccharolytic agricultural soil bacteria to oxygen. Environ. Microbiol. 12, 845–861. 10.1111/j.1462-2920.2009.02128.x 20050868

[B79] Schloss P. D. Westcott S. L. Ryabin T. Hall J. R. Hartmann M. Hollister E. B.. (2009). Introducing mothur: open-source, platform-independent, community-supported software for describing and comparing microbial communities. Appl. Environ. Microbiol. 75, 7537–7541. 10.1128/AEM.01541-09 19801464 PMC2786419

[B80] Schneckenberger K. Demin D. Stahr K. Kuzyakov Y. (2008). Microbial utilization and mineralization of ^14^C glucose added in six orders of concentration to soil. Soil Biol. Biochem. 40, 1981–1988. 10.1016/j.soilbio.2008.02.020

[B81] Six J. Frey S. D. Thiet R. K. Batten K. M. (2006). Bacterial and fungal contributions to carbon sequestration in agroecosystems. Soil Sci. Soc. Am. J. 70, 555 10.2136/sssaj2004.0347

[B82] Strickland M. S. Lauber C. Fierer N. Bradford M. A. (2009). Testing the functional significance of microbial community composition. Ecology 90, 441–451. 10.1890/08-0296.1 19323228

[B83] Treseder K. K. Balser T. C. Bradford M. A. Brodie E. L. Dubinsky E. A. Eviner V. T. (2011). Integrating microbial ecology into ecosystem models: challenges and priorities. Biogeochemistry 109, 7–18. 10.1007/s10533-011-9636-5

[B84] Verastegui Y. Cheng J. Engel K. Kolczynski D. Mortimer S. Lavigne J.. (2014). Multisubstrate isotope labeling and metagenomic analysis of active soil bacterial communities. mBio 5:e01157–14. 10.1128/mbio.01157-14 25028422 PMC4161255

[B85] Webb C. O. (2000). Exploring the phylogenetic structure of ecological communities: an example for rain forest trees. Am. Nat. 156, 145–155. 10.1086/303378 10856198

[B86] Wertz J. T. Kim E. Breznak J. A. Schmidt T. M. Rodrigues J. L. M. (2011). Genomic and physiological characterization of the *Verrucomicrobia* isolate *Diplosphaera colitermitum* gen. nov. sp. nov., reveals microaerophily and nitrogen fixation genes. Appl. Environ. Microbiol. 78, 1544–1555. 10.1128/AEM.06466-11 22194293 PMC3294482

[B87] Wieder W. R. Bonan G. B. Allison S. D. (2013). Global soil carbon projections are improved by modelling microbial processes. Nat. Clim. Change 3, 909–912. 10.1038/nclimate1951

[B88] Yan J. Hu Z. Pu Y. Brummer E. C. Ragauskas A. J. (2010). Chemical compositions of four switchgrass populations. Biomass Bioenergy 34, 48–53. 10.1016/j.biombioe.2009.09.010

[B89] Yarza P. Richter M. Peplies J. Euzeby J. Amann R. Schleifer K.-H.. (2008). The all-species living tree project: a 16S rRNA-based phylogenetic tree of all sequenced type strains. Syst. Appl. Microbiol. 31, 241–250. 10.1016/j.syapm.2008.07.001 18692976

[B90] Zak D. R. Blackwood C. B. Waldrop M. P. (2006). A molecular dawn for biogeochemistry. Trends Ecol. Evol. 21, 288–295. 10.1016/j.tree.2006.04.003 16769427

[B91] Zarda B. Hahn D. Chatzinotas A. Schönhuber W. Neef A. Amann R. I. (1997). Analysis of bacterial community structure in bulk soil by *in situ* hybridization. Arch. Microbiol. 168, 185–192. 10.1007/s002030050486

