# Supplementary material for: Unearthing the Ecology of Soil Microorganisms Using a High Resolution DNA-SIP Approach to Explore Cellulose and Xylose Metabolism in Soil
Source: Front Microbiol. 2016 May 12;7:703. doi: 10.3389/fmicb.2016.00703 (PMC4867679; doi:10.3389/fmicb.2016.00703)
Supplement: Supplementary file 1 [file Table1.PDF]

Table S1: ^{13}C-xylose responders BLAST against Living Tree Project

OTU ID	Fold change [a]	Day [b]	All days [c]	Top BLAST hits	BLAST %ID	Phylum;Class;Order
OTU.1040	4.78	1	1	*Paenibacillus daejeonensis*	100.0	*Firmicutes Bacilli Bacillales*
OTU.1069	3.85	1	1	*Paenibacillus terrigena*	100.0	*Firmicutes Bacilli Bacillales*
OTU.107	2.25	3	3	*Flavobacterium sp. 15C3*, *Flavobacterium banpakuense*	99.54	*Bacteroidetes Flavobacteria Flavobacteriales*
OTU.11	5.25	7	7	*Stenotrophomonas pavanii*, *Stenotrophomonas maltophilia*, *Pseudomonas geniculata*	99.54	*Proteobacteria Gammaproteobacteria Xanthomonadales*
OTU.131	3.07	3	3	*Flavobacterium fluvii*, *Flavobacteria bacterium HMD1033*, *Flavobacterium sp. HMD1001*	100.0	*Bacteroidetes Flavobacteria Flavobacteriales*
OTU.14	3.92	3	1, 3	*Flavobacterium oncorhynchi*, *Flavobacterium glycines*, *Flavobacterium succinicans*	99.09	*Bacteroidetes Flavobacteria Flavobacteriales*
OTU.150	3.08	14	14	No hits of at least 90% identity	86.76	*Planctomycetes Planctomycetacia Planctomycetales*
OTU.159	3.16	3	3	*Flavobacterium hibernum*	98.17	*Bacteroidetes Flavobacteria Flavobacteriales*
OTU.165	2.38	3	3	*Rhizobium skierniewicense*, *Rhizobium vignae*, *Rhizobium larrymoorei*, *Rhizobium alkalisoli*, *Rhizobium galegae*, *Rhizobium huautlense*	100.0	*Proteobacteria Alphaproteobacteria Rhizobiales*
OTU.183	3.31	3	3	No hits of at least 90% identity	89.5	*Bacteroidetes Sphingobacteriia Sphingobacteriales*
OTU.19	2.14	7	7	*Rhizobium alamii*, *Rhizobium mesosinicum*, *Rhizobium mongolense*, *Arthrobacter viscosus*, *Rhizobium sullae*, *Rhizobium yanglingense*, *Rhizobium loessense*	99.54	*Proteobacteria Alphaproteobacteria Rhizobiales*
OTU.2040	2.91	1	1	*Paenibacillus pectinilyticus*	100.0	*Firmicutes Bacilli Bacillales*
OTU.22	2.8	7	7, 14	*Paracoccus sp. NB88*	99.09	*Proteobacteria Alphaproteobacteria Rhodobacterales*
OTU.2379	3.1	3	3	*Flavobacterium pectinovorum*, *Flavobacterium sp. CS100*	97.72	*Bacteroidetes Flavobacteria Flavobacteriales*
OTU.24	2.81	7	7	*Cellulomonas aerilata*, *Cellulomonas humilata*, *Cellulomonas terrae*, *Cellulomonas soli*, *Cellulomonas xylanilytica*	100.0	*Actinobacteria Micrococcales Cellulomonadaceae*
OTU.241	3.38	3	3, 14	No hits of at least 90% identity	87.73	*Verrucomicrobia Spartobacteria Chthoniobacterales*
OTU.244	3.08	7	7	*Cellulosimicrobium funkei*, *Cellulosimicrobium terreum*	100.0	*Actinobacteria Micrococcales Promicromonosporaceae*
OTU.252	3.34	7	7	*Promicromonospora thailandica*	100.0	*Actinobacteria Micrococcales Promicromonosporaceae*
OTU.267	4.97	1	1	*Paenibacillus pabuli*, *Paenibacillus tundrae*, *Paenibacillus taichungensis*, *Paenibacillus xylanexedens*, *Paenibacillus xylanilyticus*	100.0	*Firmicutes Bacilli Bacillales*
OTU.277	3.52	3	3	*Solibius ginsengiterrae*	95.43	*Bacteroidetes Sphingobacteriia Sphingobacteriales*

Table S1 – continued from previous page

OTU ID	Fold change	Day	All days	Top BLAST hits	BLAST %ID	Phylum;Class;Order
OTU.290	3.59	1	1	*Pantoea spp.*, *Kluyvera spp.*, *Klebsiella spp.*, *Erwinia spp.*, *Enterobacter spp.*, *Buttiauxella spp.*	100.0	*Proteobacteria Gammaproteobacteria Enterobacteriales*
OTU.3	2.61	1	1	*[Brevibacterium] frigoritolerans*, *Bacillus sp. LMG 20238*, *Bacillus coahuilensis m4-4*, *Bacillus simplex*	100.0	*Firmicutes Bacilli Bacillales*
OTU.319	3.98	1	1	*Paenibacillus xinjiangensis*	97.25	*Firmicutes Bacilli Bacillales*
OTU.32	3.0	3	3, 7, 14	*Sandaracinus amylolyticus*	94.98	*Proteobacteria Deltaproteobacteria Myxococcales*
OTU.335	2.53	1	1	*Paenibacillus thailandensis*	98.17	*Firmicutes Bacilli Bacillales*
OTU.346	3.44	3	3	*Pseudoduganella violaceinigra*	99.54	*Proteobacteria Betaproteobacteria Burkholderiales*
OTU.3507	2.36	1	1	*Bacillus spp.*	98.63	*Firmicutes Bacilli Bacillales*
OTU.3540	2.52	3	3	*Flavobacterium terrigena*	99.54	*Bacteroidetes Flavobacteria Flavobacteriales*
OTU.360	2.98	3	3	*Flavisolibacter ginsengisoli*	95.0	*Bacteroidetes Sphingobacteriia Sphingobacteriales*
OTU.369	5.05	1	1	*Paenibacillus sp. D75*, *Paenibacillus glycanilyticus*	100.0	*Firmicutes Bacilli Bacillales*
OTU.37	2.68	7	7	*Phycicola gilvus*, *Microterricola viridarii*, *Frigoribacterium faeni*, *Frondihabitans sp. RS-15*, *Frondihabitans australicus*	100.0	*Actinobacteria Micrococcales Microbacteriaceae*
OTU.394	4.06	1	1	*Paenibacillus pocheonensis*	100.0	*Firmicutes Bacilli Bacillales*
OTU.4	2.84	7	7, 14	*Agromyces ramosus*	100.0	*Actinobacteria Micrococcales Microbacteriaceae*
OTU.4446	3.49	7	7	*Catenuloplanes niger*, *Catenuloplanes castaneus*, *Catenuloplanes atrovinosus*, *Catenuloplanes crispus*, *Catenuloplanes nepalensis*, *Catenuloplanes japonicus*	97.72	*Actinobacteria Frankiales Nakamurellaceae*
OTU.4743	2.24	1	1	*Lysinibacillus fusiformis*, *Lysinibacillus sphaericus*	99.09	*Firmicutes Bacilli Bacillales*
OTU.48	2.99	1	1, 3	*Aeromonas spp.*	100.0	*Proteobacteria Gammaproteobacteria aaa34a10*
OTU.5	3.69	7	7	*Delftia tsuruhatensis*, *Delftia lacustris*	100.0	*Proteobacteria Betaproteobacteria Burkholderiales*
OTU.5284	3.56	7	7	*Isoptericola nanjingensis*, *Isoptericola hypogeus*, *Isoptericola variabilis*	98.63	*Actinobacteria Micrococcales Promicromonosporaceae*
OTU.5603	3.96	1	1	*Paenibacillus uliginis*	100.0	*Firmicutes Bacilli Bacillales*
OTU.57	4.39	1	1, 3, 7, 14, 30	*Paenibacillus castaneae*	98.62	*Firmicutes Bacilli Bacillales*
OTU.5906	3.16	3	3	*Terrimonas sp. M-8*	96.8	*Bacteroidetes Sphingobacteriia Sphingobacteriales*
OTU.6	3.24	3	3	*Cellvibrio fulvus*	100.0	*Proteobacteria Gammaproteobacteria Pseudomonadales*

Table S1 – continued from previous page

**OTU ID**	**Fold change**	**Day**	**All days**	**Top BLAST hits**	**BLAST %ID**	**Phylum;Class;Order**
OTU.62	2.57	7	7	*Nakamurella flavida*	100.0	*Actinobacteria Frankiales Nakamurellaceae*
OTU.6203	3.32	3	3	*Flavobacterium granuli*, *Flavobacterium glaciei*	100.0	*Bacteroidetes Flavobacteria Flavobacteriales*
OTU.68	3.74	7	7	*Shigella flexneri*, *Escherichia fergusonii*, *Escherichia coli*, *Shigella sonnei*	100.0	*Proteobacteria Gammaproteobacteria Enterobacteriales*
OTU.760	2.89	3	3	*Dyadobacter hamtensis*	98.63	*Bacteroidetes Cytophagia Cytophagales*
OTU.8	2.26	1	1	*Bacillus niacini*	100.0	*Firmicutes Bacilli Bacillales*
OTU.843	3.62	1	1	*Paenibacillus agarexedens*	100.0	*Firmicutes Bacilli Bacillales*
OTU.9	2.04	1	1	*Bacillus megaterium*, *Bacillus flexus*	100.0	*Firmicutes Bacilli Bacillales*

[a] Maximum observed log_2 of fold change.
[b] Day of maximum fold change.
[c] All response days.
