# Supplementary material for: Unearthing the Ecology of Soil Microorganisms Using a High Resolution DNA-SIP Approach to Explore Cellulose and Xylose Metabolism in Soil
Source: Front Microbiol. 2016 May 12;7:703. doi: 10.3389/fmicb.2016.00703 (PMC4867679; doi:10.3389/fmicb.2016.00703)
Supplement: Supplementary file 2 [file Table2.PDF]

Table S2: ^{13}C-cellulose responders BLAST against Living Tree Project

OTU ID	Fold change [a]	Day [b]	All days [c]	Top BLAST hits	BLAST %ID	Phylum;Class;Order
OTU.100	2.66	14	14	*Pseudoxanthomonas sacheonensis*, *Pseudoxanthomonas dokdonensis*	100.0	*Proteobacteria Gammaproteobacteria Xanthomonadales*
OTU.1023	4.61	30	30	No hits of at least 90% identity	80.54	*Verrucomicrobia Spartobacteria Chthoniobacterales*
OTU.1065	5.31	14	14, 30	No hits of at least 90% identity	84.55	*Planctomycetes Planctomycetacia Planctomycetales*
OTU.1087	4.32	14	14, 30	*Devosia soli*, *Devosia crocina*, *Devosia riboflavina*	99.09	*Proteobacteria Alphaproteobacteria Rhizobiales*
OTU.1094	3.69	30	30	*Sporocytophaga myxococcoides*	99.55	*Bacteroidetes Cytophagia Cytophagales*
OTU.11	3.41	14	14	*Stenotrophomonas pavanii*, *Stenotrophomonas maltophilia*, *Pseudomonas geniculata*	99.54	*Proteobacteria Gammaproteobacteria Xanthomonadales*
OTU.114	2.78	14	14	*Herbaspirillum sp. SUEMI03*, *Herbaspirillum sp. SUEMI10*, *Oxalicibacterium solurbis*, *Herminiimonas fonticola*, *Oxalicibacterium horti*	100.0	*Proteobacteria Betaproteobacteria Burkholderiales*
OTU.119	3.31	14	14, 30	*Brevundimonas alba*	100.0	*Proteobacteria Alphaproteobacteria Caulobacterales*
OTU.120	4.76	14	14, 30	*Vampirovibrio chlorellavorus*	94.52	*Cyanobacteria SM1D11 uncultured-bacterium*
OTU.1204	4.32	30	30	*Planctomyces limnophilus*	91.78	*Planctomycetes Planctomycetacia Planctomycetales*
OTU.1312	4.07	30	30	*Paucimonas lemoignei*	99.54	*Proteobacteria Betaproteobacteria Burkholderiales*
OTU.132	2.81	14	14	*Streptomyces spp.*	100.0	*Actinobacteria Streptomycetales Streptomycetaceae*
OTU.150	4.06	14	14	No hits of at least 90% identity	86.76	*Planctomycetes Planctomycetacia Planctomycetales*
OTU.1533	3.43	30	30	No hits of at least 90% identity	82.27	*Verrucomicrobia Spartobacteria Chthoniobacterales*
OTU.154	3.24	14	14	*Pseudoxanthomonas mexicana*, *Pseudoxanthomonas japonensis*	100.0	*Proteobacteria Gammaproteobacteria Xanthomonadales*
OTU.165	3.1	14	14	*Rhizobium skierniewicense*, *Rhizobium vignae*, *Rhizobium larrymoorei*, *Rhizobium alkalisoli*, *Rhizobium galegae*, *Rhizobium huautlense*	100.0	*Proteobacteria Alphaproteobacteria Rhizobiales*
OTU.1754	4.48	14	14	*Asticcacaulis biprosthecium*, *Asticcacaulis benevestitus*	96.8	*Proteobacteria Alphaproteobacteria Caulobacterales*
OTU.185	4.37	14	14, 30	No hits of at least 90% identity	85.14	*Verrucomicrobia Spartobacteria Chthoniobacterales*
OTU.19	2.44	14	14	*Rhizobium alamii*, *Rhizobium mesosinicum*, *Rhizobium mongolense*, *Arthrobacter viscosus*, *Rhizobium sullae*, *Rhizobium yanglingense*, *Rhizobium loessense*	99.54	*Proteobacteria Alphaproteobacteria Rhizobiales*

Table S2 – continued from previous page

OTU ID	Fold change	Day	All days	Top BLAST hits	BLAST %ID	Phylum;Class;Order
OTU.2192	3.49	30	14, 30	No hits of at least 90% identity	83.56	*Verrucomicrobia Spartobacteria Chthoniobacterales*
OTU.228	2.54	30	30	*Sorangium cellulosum*	98.17	*Proteobacteria Deltaproteobacteria Myxococcales*
OTU.241	2.66	14	14	No hits of at least 90% identity	87.73	*Verrucomicrobia Spartobacteria Chthoniobacterales*
OTU.257	2.94	14	14	*Lentzea waywayandensis*, *Lentzea flaviverrucosa*	100.0	*Actinobacteria Pseudonocardiales Pseudonocardiaceae*
OTU.266	4.54	30	14, 30	No hits of at least 90% identity	83.64	*Verrucomicrobia Spartobacteria Chthoniobacterales*
OTU.28	2.59	14	14	*Rhizobium giardinii*, *Rhizobium tubonense*, *Rhizobium tibeticum*, *Rhizobium mesoamericanum CCGE 501*, *Rhizobium herbae*, *Rhizobium endophyticum*	99.54	*Proteobacteria Alphaproteobacteria Rhizobiales*
OTU.285	3.55	30	14, 30	*Blastopirellula marina*	90.87	*Planctomycetes Planctomycetacia Planctomycetales*
OTU.32	2.34	3	3	*Sandaracinus amylolyticus*	94.98	*Proteobacteria Deltaproteobacteria Myxococcales*
OTU.327	2.99	14	14	*Asticcacaulis biprosthecium*, *Asticcacaulis benevestitus*	98.63	*Proteobacteria Alphaproteobacteria Caulobacterales*
OTU.351	3.54	14	14, 30	*Pirellula staleyi DSM 6068*	91.86	*Planctomycetes Planctomycetacia Planctomycetales*
OTU.3594	3.83	30	30	*Chondromyces robustus*	90.41	*Proteobacteria Deltaproteobacteria Myxococcales*
OTU.3775	3.88	14	14	*Devosia glacialis*, *Devosia chinhatensis*, *Devosia geojensis*, *Devosia yakushimensis*	98.63	*Proteobacteria Alphaproteobacteria Rhizobiales*
OTU.429	3.7	30	14, 30	*Devosia limi*, *Devosia psychrophila*	97.72	*Proteobacteria Alphaproteobacteria Rhizobiales*
OTU.4322	4.19	14	7, 14, 30	No hits of at least 90% identity	89.14	*Chloroflexi Herpetosiphonales Herpetosiphonaceae*
OTU.442	3.05	30	30	*Chondromyces robustus*	92.24	*Proteobacteria Deltaproteobacteria Myxococcales*
OTU.465	3.79	30	30	*Ohtaekwangia kribbensis*	92.73	*Bacteroidetes Cytophagia Cytophagales*
OTU.473	3.58	14	14	*Pirellula staleyi DSM 6068*	90.91	*Planctomycetes Planctomycetacia Planctomycetales*
OTU.484	4.92	14	14, 30	No hits of at least 90% identity	89.09	*Planctomycetes Planctomycetacia Planctomycetales*
OTU.5	2.69	14	14	*Delftia tsuruhatensis*, *Delftia lacustris*	100.0	*Proteobacteria Betaproteobacteria Burkholderiales*
OTU.518	4.8	14	14	*Hydrogenophaga intermedia*	100.0	*Proteobacteria Betaproteobacteria Burkholderiales*
OTU.5190	3.6	30	14, 30	No hits of at least 90% identity	88.13	*Chloroflexi Herpetosiphonales Herpetosiphonaceae*
OTU.541	4.49	30	30	No hits of at least 90% identity	84.23	*Verrucomicrobia Spartobacteria Chthoniobacterales*
OTU.5539	4.01	14	14	*Devosia subaequoris*	98.17	*Proteobacteria Alphaproteobacteria Rhizobiales*
OTU.573	3.03	30	30	*Adhaeribacter aerophilus*	92.76	*Bacteroidetes Cytophagia Cytophagales*

Table S2 – continued from previous page

OTU ID	Fold change	Day	All days	Top BLAST hits	BLAST %ID	Phylum;Class;Order
OTU.6	3.62	7	3, 7, 14	*Cellvibrio fulvus*	100.0	*Proteobacteria Gammaproteobacteria Pseudomonadales*
OTU.600	3.48	30	30	No hits of at least 90% identity	80.37	*Planctomycetes Planctomycetacia Planctomycetales*
OTU.6062	4.83	30	30	*Dokdonella sp. DC-3*, *Luteibacter rhizovicinus*	97.26	*Proteobacteria Gammaproteobacteria Xanthomonadales*
OTU.627	4.43	14	14	*Verrucomicrobiaceae bacterium DC2a-G7*	100.0	*Verrucomicrobia Verrucomicrobiae Verrucomicrobiales*
OTU.633	3.84	30	30	No hits of at least 90% identity	89.5	*Proteobacteria Deltaproteobacteria Myxococcales*
OTU.638	4.0	30	30	*Luteolibacter sp. CCTCC AB 2010415*, *Luteolibacter algae*	93.61	*Verrucomicrobia Verrucomicrobiae Verrucomicrobiales*
OTU.64	4.31	14	7, 14, 30	No hits of at least 90% identity	89.5	*Chloroflexi Herpetosiphonales Herpetosiphonaceae*
OTU.663	3.63	30	30	*Pirellula staleyi DSM 6068*	90.87	*Planctomycetes Planctomycetacia Planctomycetales*
OTU.669	3.34	30	30	*Ohtaekwangia koreensis*	92.69	*Bacteroidetes Cytophagia Cytophagales*
OTU.670	2.87	30	30	*Adhaeribacter aerophilus*	91.78	*Bacteroidetes Cytophagia Cytophagales*
OTU.766	3.21	14	14, 30	*Devosia insulae*	99.54	*Proteobacteria Alphaproteobacteria Rhizobiales*
OTU.83	5.61	14	7, 14, 30	*Luteolibacter sp. CCTCC AB 2010415*	97.72	*Verrucomicrobia Verrucomicrobiae Verrucomicrobiales*
OTU.862	5.87	14	14	*Allokutzneria albata*	100.0	*Actinobacteria Pseudonocardiales Pseudonocardiaceae*
OTU.899	2.28	30	30	*Enhygromyxa salina*	97.72	*Proteobacteria Deltaproteobacteria Myxococcales*
OTU.90	2.94	14	14, 30	*Sphingopyxis panaciterrae*, *Sphingopyxis chilensis*, *Sphingopyxis sp. BZ30*, *Sphingomonas sp.*	100.0	*Proteobacteria Alphaproteobacteria Sphingomonadales*
OTU.900	4.87	14	14	*Brevundimonas vesicularis*, *Brevundimonas nasdae*	100.0	*Proteobacteria Alphaproteobacteria Caulobacterales*
OTU.971	3.68	30	30	No hits of at least 90% identity	78.57	*Chloroflexi Anaerolineae Anaerolineales*
OTU.98	3.68	14	7, 14, 30	No hits of at least 90% identity	88.18	*Chloroflexi Herpetosiphonales Herpetosiphonaceae*
OTU.982	4.47	14	14	*Devosia neptuniae*	100.0	*Proteobacteria Alphaproteobacteria Rhizobiales*

[a] Maximum observed log_2 of fold change.
[b] Day of maximum fold change.
[c] All response days.
