# Supplementary material for: Unearthing the Ecology of Soil Microorganisms Using a High Resolution DNA-SIP Approach to Explore Cellulose and Xylose Metabolism in Soil
Source: Front Microbiol. 2016 May 12;7:703. doi: 10.3389/fmicb.2016.00703 (PMC4867679; doi:10.3389/fmicb.2016.00703)
Supplement: Supplementary file 3 [file Presentation1.pdf]

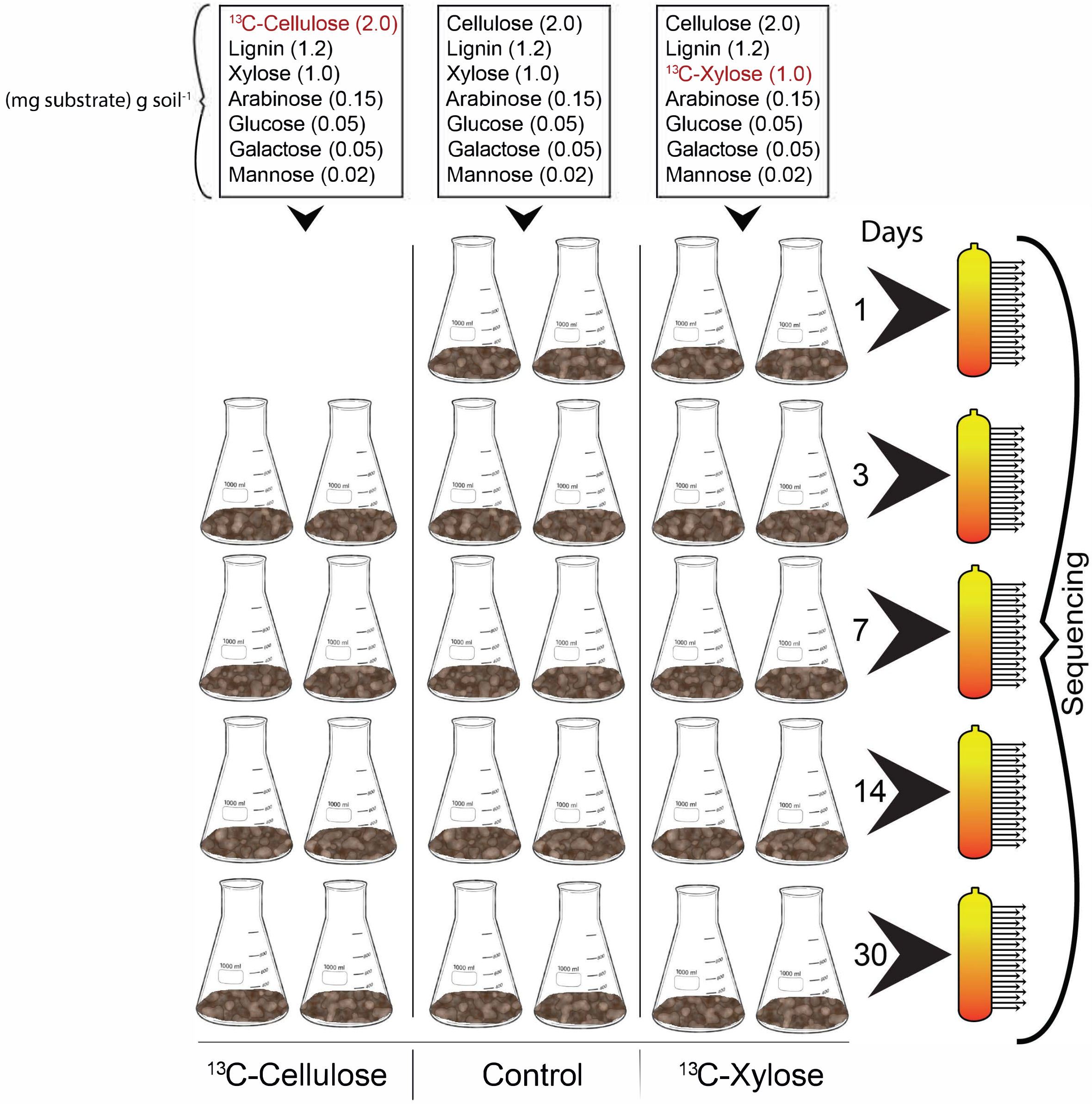

Figure S1

We added a carbon mixture that contained inorganic salts and amino acids (not shown here) to each soil microcosm where the only difference between treatments was the ^{13}C-labeled isotope (in red). At days 1, 3, 7, 14, and 30 replicate microcosms were destructively harvested for downstream molecular applications. DNA from each treatment and time (n = 14) was subjected to CsCl density gradient centrifugation and density gradients were fractionated (orange tubes wherein each arrow represents a fraction from the density gradient). SSU rRNA genes from each gradient fraction were PCR amplified and sequenced. In addition, SSU rRNA genes were also PCR amplified and sequenced from non-fractionated DNA to represent the soil microbial community.

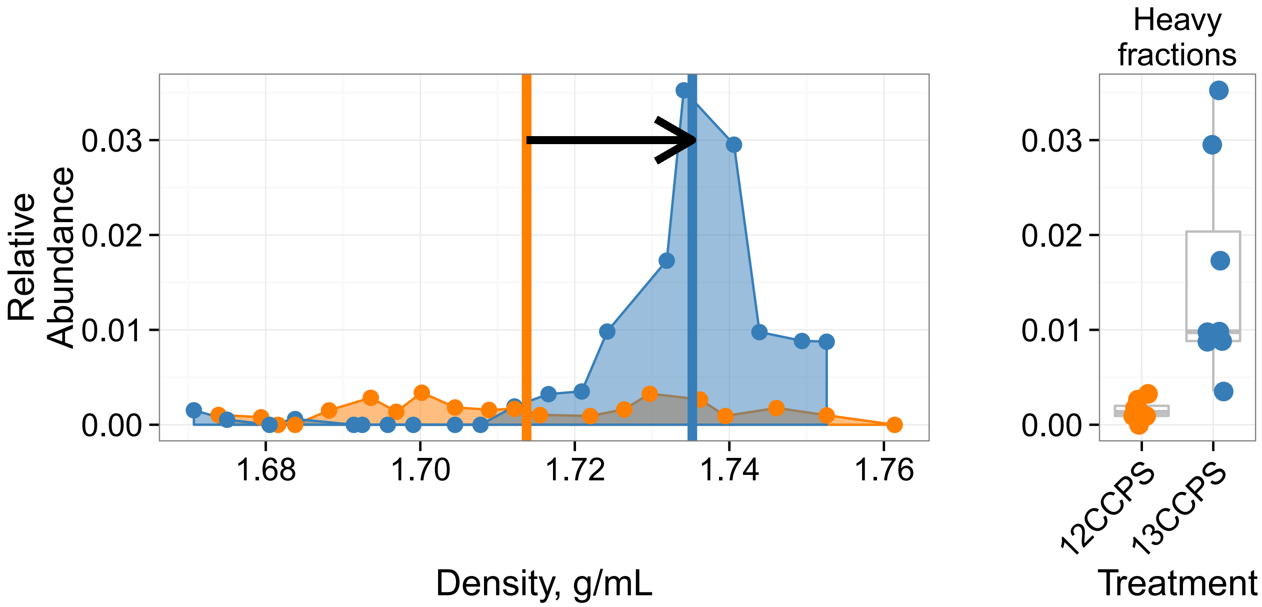

Figure S2
Density profile for a single cellulose responder in the ^{13}C-cellulose treatment (blue) and control (orange). Vertical lines show center of mass for each density profile and the arrow denotes the magnitude and direction of $\Delta(\widehat{BD})$. Right panel shows relative abundance values in the high density fractions (The boxplot line is the median value). The box spans one interquartile range (IR) about the median, whiskers extend 1.5 times the IR.

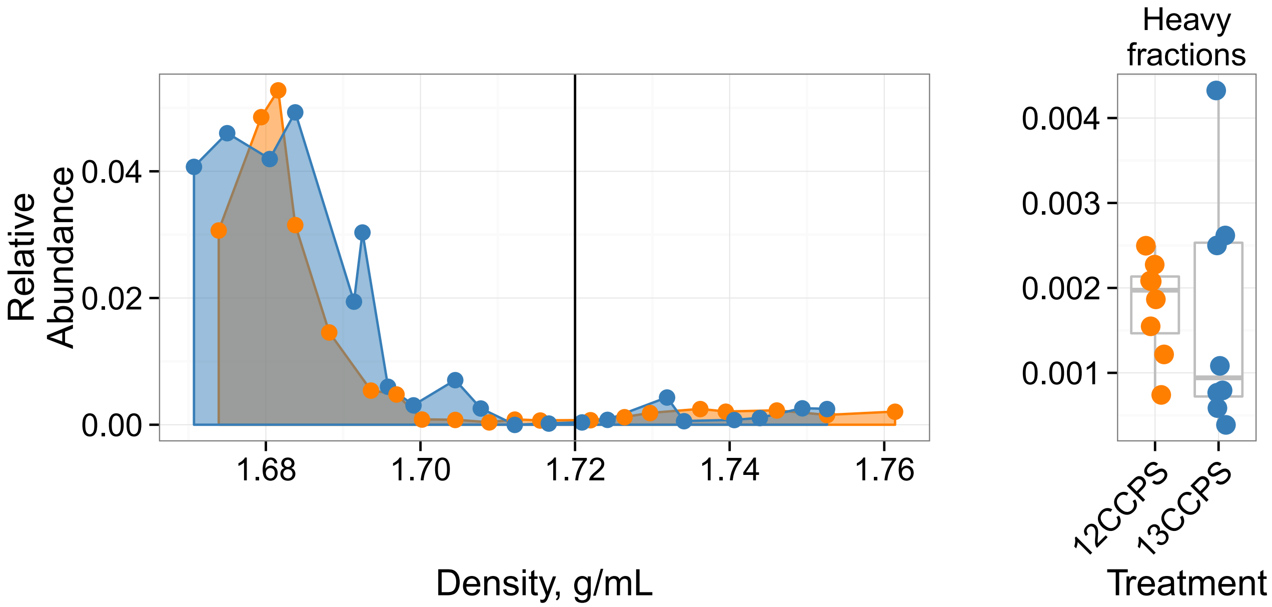

Figure S3

Density profile for a single non-responder OTU. The ^{13}C-cellulose treatment is in blue and the control treatment is in orange. The vertical line shows where high density fractions begin as defined in our analysis. The right panel shows relative abundance values in the high density fractions for each gradient (The boxplot line is the median value). The box spans one interquartile range (IR) about the median, whiskers extend 1.5 times the IR.

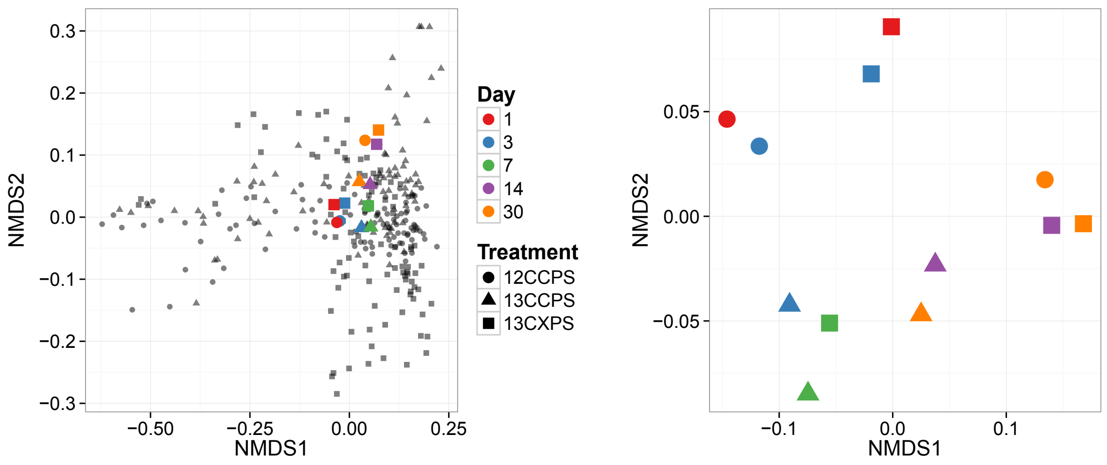

Figure S4
NMDS analysis of SSU rRNA gene composition in non-fractionated DNA (colored points) indicates that isotopic labeling does not alter overall microbial community composition, microbial community composition in the soil microcosms changes over time, and variance in non-fractionated DNA is smaller than variance in fractionated DNA (black points). SSU rRNA gene sequences were determined for non-fractionated DNA from the unlabeled control, ^{13}C-xylose, and ^{13}C-cellulose treatments over time (colors indicate time, different symbols used for different treatments). Distance in SSU rRNA gene composition was quantified with the weighted UniFrac metric. The leftmost panel indicates NMDS of data from both non-fractionated and fractionated samples. The rightmost panel indicates NMDS of data only from non-fractionated DNA. Statistical analysis is presented in main text. Samples not represented in the ordination did not sequence successfully and constitute missing data (e.g. "12CCPS" day 7).

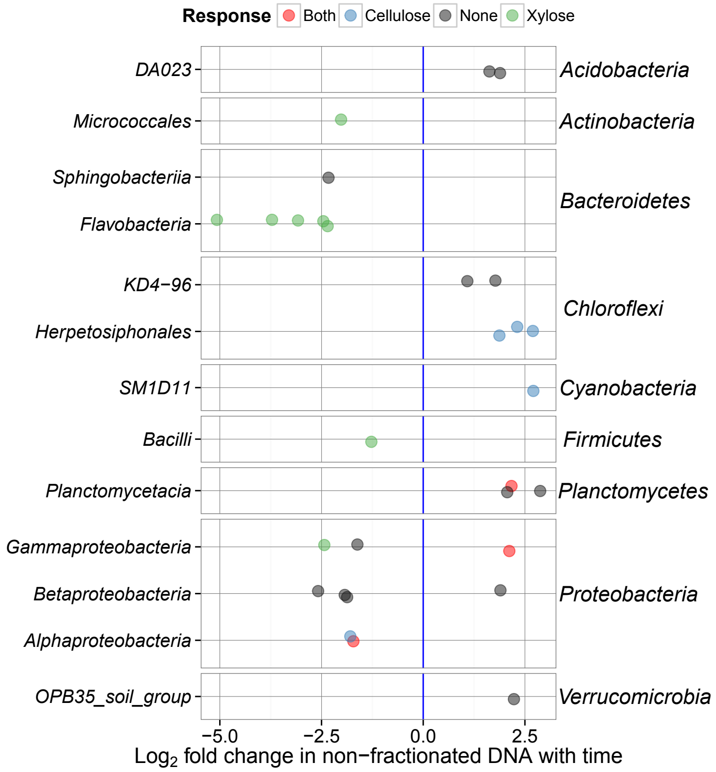

Figure S5

Change in non-fractionated DNA relative abundance versus time (expressed as LFC) for OTUs that changed significantly over time (P-value $<$ 0.10, Wald test). Each panel shows one phylum (labeled on the right). The taxonomic class is indicated on the left. OTUs that responded to just xylose are shown in green, just cellulose in blue, and both xylose and cellulose in red.

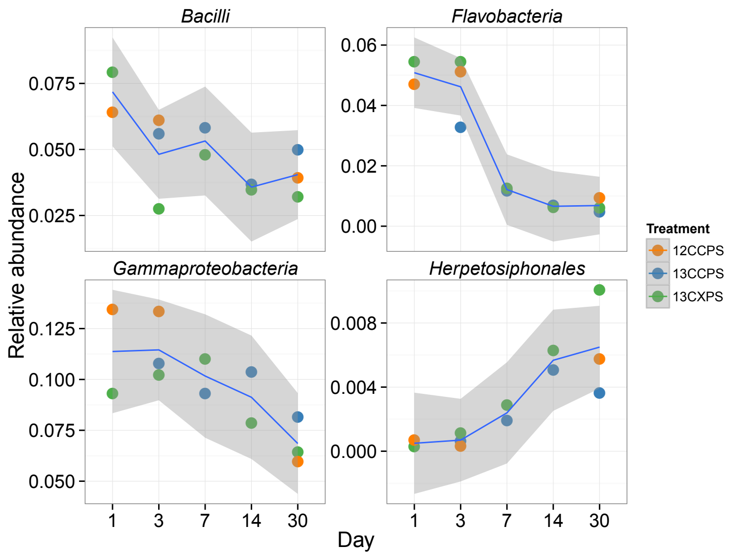

Figure S6

Relative abundance in non-fractionated DNA versus time for classes that changed significantly. Samples from different treatments are labeled with different colors as indicated in the scale. Statistical analysis is presented in main text.

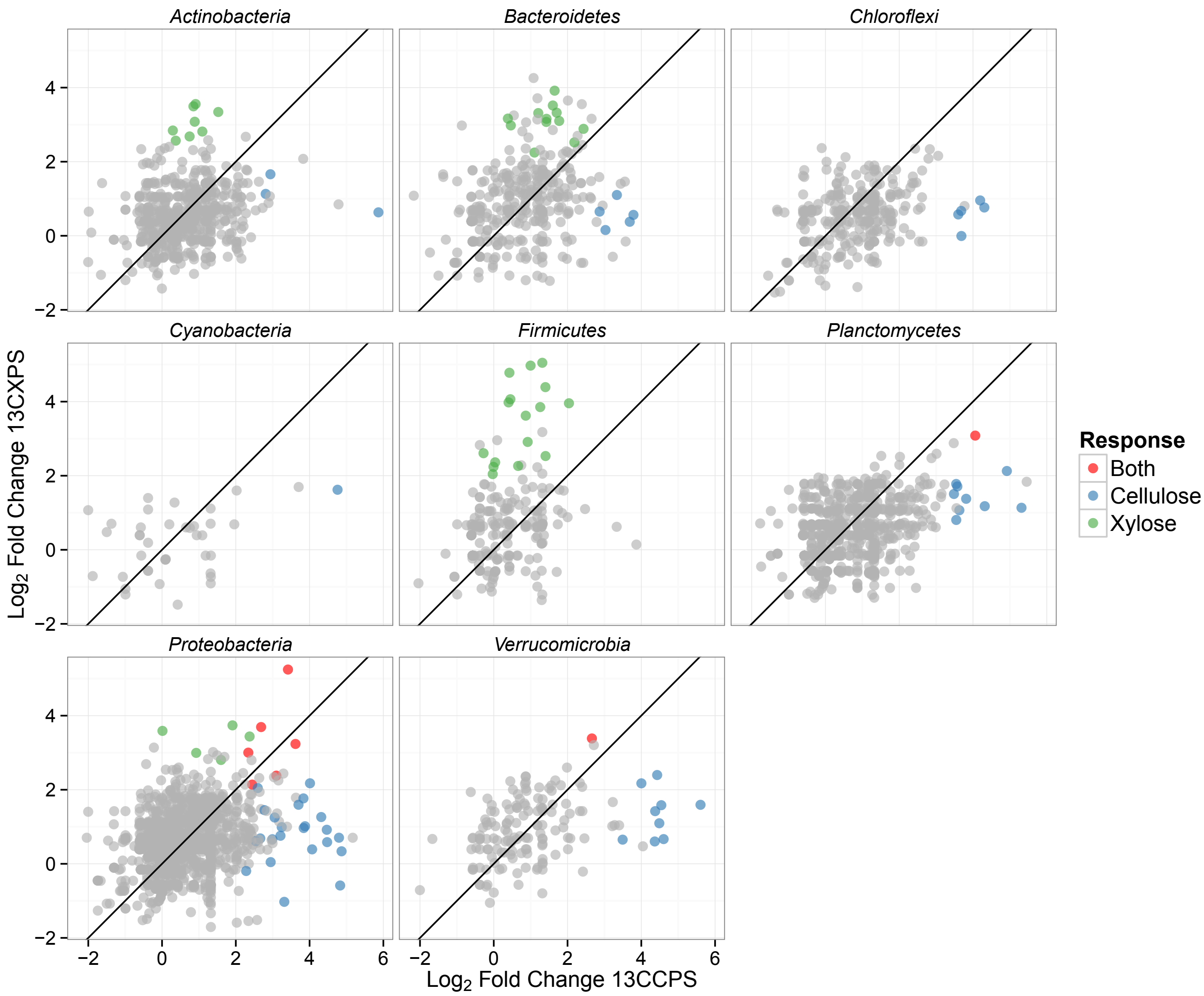

Figure S7

Maximum enrichment at any point in time in high density fractions of 13C-treatments relative to control (expressed as LFC) shown for ^{13}C-cellulose versus ^{13}C-xylose treatments. Each point represents an OTU. Blue points are cellulose responders, green xylose responders, red are responders to both xylose and cellulose, and gray points are OTUs that did not respond to either substrate. Line indicates a slope of one.

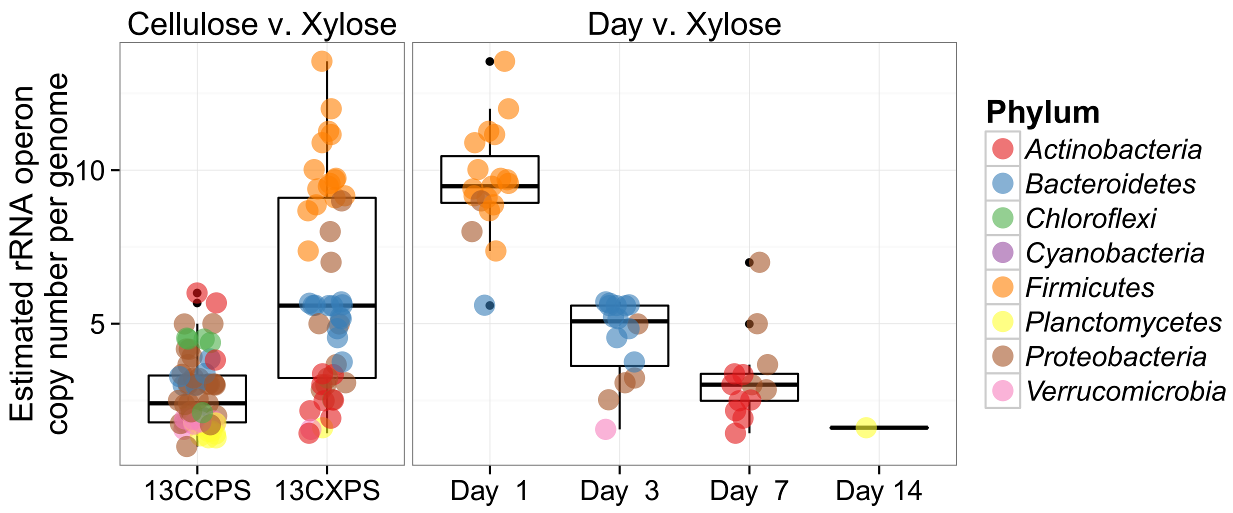

Figure S8
Estimated rrn copy number for xylose and cellulose responders. The leftmost panel contrasts estimated rrn copy number for cellulose (13CCPS) and xylose (13CXPS) responders. The right panel shows estimated rrn copy number versus time of first response for xylose responders. Colors denote the phylum of the OTUs (see legend).
